# Intracellular antibody targeting HBx suppresses invasion and metastasis in hepatitis B virus‐related hepatocarcinogenesis via protein phosphatase 2A‐B56γ‐mediated dephosphorylation of protein kinase B

**DOI:** 10.1111/cpr.13304

**Published:** 2022-07-10

**Authors:** Lin Che, Ze‐Bang Du, Wei‐Hua Wang, Jia‐Shen Wu, Tun Han, Yuan‐Yuan Chen, Pei‐Yu Han, Zhao Lei, Xiao‐Xuan Chen, Yun He, Ling Xu, Xu Lin, Zhong‐Ning Lin, Yu‐Chun Lin

**Affiliations:** ^1^ State Key Laboratory of Molecular Vaccinology and Molecular Diagnostics, School of Public Health Xiamen University Xiamen China; ^2^ China CDC Key Laboratory of Environment and Population Health National Institute of Environmental Health, Chinese Center for Disease Control and Prevention Beijing China; ^3^ Wuxi School of Medicine Jiangnan University Wuxi China; ^4^ Key Laboratory of Ministry of Education for Gastrointestinal Cancer, School of Basic Medical Sciences Fujian Medical University Fuzhou China

## Abstract

**Objectives:**

Hepatitis B virus X (HBx) is closely associated with HBV‐related hepatocarcinogenesis via the inactivation of tumour suppressors. Protein phosphatase 2A (PP2A) regulatory subunit B56 gamma (B56γ), as a tumour suppressor, plays a critical role in regulating cellular phosphorylation signals via dephosphorylation of signalling proteins. However, the underlying mechanism that B56γ involved in regulating HBx‐associated hepatocarcinogenesis phenotypes and mediating anti‐HBx antibody‐mediated tumour suppression remains unknown.

**Materials and Methods:**

We used bioinformatics analysis, paired HCC patient specimens, HBx transgenic (HBx‐Tg) mice, xenograft nude mice, HBV stable replication in the HepG2.2.15 cells, and anti‐HBx antibody intervention to systematically evaluate the biological function of protein kinase B (AKT) dephosphorylation through B56γ in HBx‐associated hepatocarcinogenesis.

**Results:**

Bioinformatics analysis revealed that AKT, matrix metalloproteinase 2 (MMP2), and MMP9 were markedly upregulated, while cell migration and viral carcinogenesis pathways were activated in HBV‐infected liver tissues and HBV‐associated HCC tissues. Our results demonstrated that HBx‐expression promotes AKT phosphorylation (p‐AKT^Thr308/Ser473^), mediating the migration and invasion phenotypes in vivo and in vitro. Importantly, in clinical samples, HBx and B56γ were downregulated in HBV‐associated HCC tumour tissues compared with peritumor tissues. Moreover, intervention with site‐directed mutagenesis (AKT^T308A^, AKT^S473A^) of p‐AKT^Thr308/Ser473^ mimics dephosphorylation, genetics‐based B56γ overexpression, and intracellular anti‐HBx antibody inhibited cell growth, migration, and invasion in HBx‐expressing HCC cells.

**Conclusions:**

Our results demonstrated that B56γ inhibited HBV/HBx‐dependent hepatocarcinogenesis by regulating the dephosphorylation of p‐AKT^Thr308/Ser473^ in HCC cells. The intracellular anti‐HBx antibody and the activator of B56γ may provide a multipattern chemopreventive strategy against HBV‐related HCC.

## Graphical abstract

Schematic diagram of PP2A‐B56γ mediated the dephosphorylation of p‐AKT^Thr308/Ser473^ in HBx‐expressing HCC cells to regulate the migration and invasion phenotypes of HBV/HBx‐related hepatocarcinogenesis. In current study, HBx‐expression induced the phosphorylation of specific AKT sites (p‐AKT^Thr308/Ser473^) involved in mediating the migration and invasion phenotypes of HCC cells. The inducible upregulation of B56γ mediated the dephosphorylation of p‐AKT^Thr308/Ser473^ in HBx‐expressing HCC cells. Specific blockade of HBx‐expression via pTT5‐anti‐HBx plasmid‐mediated targeting intracellular anti‐HBx mAb production and genetic activation of B56γ would help to target the p‐AKT^Thr308/Ser473^‐MMP2/9 signalling axis to mediate the multipattern chemoprevention and intervention in HBV/HBx‐related hepatocarcinogenesis.

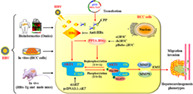

## 1 INTRODUCTION

Hepatocellular carcinoma (HCC) is one of the most common malignancies and ranks the third leading cause of cancer‐related deaths worldwide.^1^ Unfortunately, available HCC therapies are effective in a small minority of people. In addition to traditional surgery and chemotherapy, immunotherapy has been at the forefront of research into the personalized treatment of HCC. Other therapeutics, such as vaccines, oncolytic viruses, and monoclonal antibodies, are emerging for prolonging and improving patients' quality of life with advanced HCC.^2^, ^3^ It is well known that chronic hepatitis B virus (HBV) infection contributes to hepatocarcinogenesis and its outcomes, while chronic HBV infection is responsible for 50%–80% of HCC cases worldwide.^4^, ^5^, ^6^ HBV X protein (HBx), a multifunctional protein encoded by the HBV *X* gene, is likely to be involved in several steps of the development and progression of hepatocarcinogenesis, including the characterized phenotype of intrahepatic metastases in HBV‐related HCC.^7^, ^8^ Therefore, there is a need for better understanding of the molecular mechanisms of how HBx mediates hepatocarcinogenesis, which will be critical for more effective interventional and therapeutic targets.

Protein phosphatase 2A (PP2A), a tumour suppressor with dephosphorylation function following activation, is assembled of three subunits (A, B, and C) in a holoenzyme. The regulatory B subunit can be grouped into four families with 16 isoforms.^9^, ^10^ Xi et al. suggested that PP2A regulatory subunit B56 could increase HBV core protein C‐terminal domain dephosphorylation and decrease nuclear HBV episomes, thereby inhibiting multiple stages of HBV replication.^11^ Recently, we also found that B56γ could promote p53/p21 pathway‐dependent cell cycle arrest, resulting in apoptosis of HBx‐expressing hepatic cells through dephosphorylating p‐p53^Thr55^, suggesting that B56γ could be a therapeutic target for HBV‐related hepatic injury.^12^ Nevertheless, the molecular mechanism by which serine/threonine (Ser/Thr) dephosphorylation of targeted substrates regulates HBV‐induced HCC invasion and metastasis phenotype remains unclear.

While many kinases govern Ser/Thr phosphorylation, its dephosphorylation is regulated by only a few phosphatases.^13^, ^14^ Known as protein kinase B (a Ser/Thr protein kinase), AKT's phosphorylation is involved in cell proliferation, migration, and metabolism via regulating signal transduction pathways.^15^ It has been reported that PP2A can negatively regulate tumour progression by regulating the dephosphorylation of AKT. Indeed, Umesalma et al. showed that reactivation of endogenous PP2A with a small‐molecule activator of PP2A (SMAP) reduced p‐AKT^Ser473^ in a novel RAB‐like GTPase RABL6A‐dependent manner and blocked the growth of neuroendocrine tumour cells.^16^ Furthermore, amphetamine can inhibit phosphorylation of p‐AKT^Thr308/Ser473^ by increasing the activity of PP2A.^17^ Recent studies have shown that B56γ is an integral component of the human T‐cell leukaemia virus type‐1 (HTLV‐1) intasome, which plays an important role in HTLV‐1 infection, suggesting that B56γ is a potential biomarker and target for antiviral therapy.^18^ However, it remains unclear whether B56γ can regulate dephosphorylation of AKT to regulate HBx‐associated hepatocarcinogenesis is not fully understood.

In this study, we first provided evidence that B56γ‐dependent dephosphorylation of p‐AKT^Thr308/Ser473^ participated in the HBx‐expression cells migration and invasion phenotypes, while genetic activation of B56γ suppressed the HBV/HBx‐associated hepatocarcinogenesis. Furthermore, we first adopted an anti‐HBx cell‐penetrating antibody to suppress hepatocarcinogenesis phenotypes via targeting blockade of intracellular HBx. Thus, our findings demonstrated that the B56γ/p‐AKT^Thr308/Ser473^ signalling axis is a post‐translational modification (PTM) mechanism that could potentially target for the multipattern chemoprevention and intervention in environmental hepatocarcinogenesis.

## 2 MATERIALS AND METHODS

### 2.1 Gene Expression Omnibus data analysis

Microarray data (GSE83148) and RNA sequencing (GSE94660) on mRNA expression were screened from the Gene Expression Omnibus (GEO) database (http://www.ncbi.nlm.nih.gov/geo/). Bioinformatics analysis was performed as described previously.^19^ Briefly, volcano plots and heatmaps were drawn based on the obtained differential expression genes (DEGs). Gene ontology (GO) and Kyoto Encyclopedia of Genes and Genomes (KEGG) pathway enrichment analyses were performed to identify pathways significantly affected by the hepatocarcinogenesis process of DEGs. Gene set enrichment analysis (GSEA) was used to identify signalling pathways that are differentially activated between HBV‐positive infected HCC tumour tissues and their adjacent paired non‐neoplastic liver tissues.

### 2.2 Bioinformatics analysis of AKT and its phosphorylation modification

The amino acid sequences of AKT in different species were blasted through the Uniprot website (https://www.uniprot.org/) to screen the functional domains. The phosphorylation sites of AKT were predicted with PhosphoNET, NetPhos 3.1 Server, and the DISPHOS1.3 website. The conserved phosphorylation sites of AKT were analysed at the Weblogo website (http://weblogo.berkeley.edu/logo.cgi).

### 2.3 Tissue specimens from HCC patients

HCC tumour tissues and corresponding adjacent peritumor tissues were obtained from 22 patients who had a positive serum hepatitis B surface antigen (HBsAg) status in the Affiliated Zhongshan Hospital of Xiamen University. All participants provided written informed consent, and the research was approved by the Medical Ethics Committee of Xiamen University. The levels of B56γ and HBx were detected by Western blot (WB), haematoxylin and eosin (HE), and immunohistochemistry (IHC) analysis.

### 2.4 HBx transgenic (HBx‐Tg) mice study

The HBx‐Tg mice were constructed as mentioned in our previous study.^12^ At 12‐month‐old, the C57BL/6 wide type (WT) and HBx‐Tg mice were sacrificed, and the livers were collected to extract tissue lysates and make serial sections. The protein expression levels were detected by WB, and the colocalization and distribution of HBx and p‐AKT^Thr308/Ser473^ or MMP2/MMP9 were analysed by immunofluorescence (IF) analysis. All animal experiments were approved by the Experimental Animal Ethics Committee of Xiamen University.

### 2.5 Xenograft tumour study

BALB/c nude mice (4–6 weeks old) were obtained from the SLAC Laboratory Animal Co., Ltd. (Shanghai, China). To explore the effect of HBx expression and *PPP2R5C* (*2R5C*) genetic targeting intervention on the growth of HCC cells in xenograft tumours and tumour metastasis study, an HBV genome‐integrated and highly metastatic MHCC97H cell line was employed to inoculate into BALB/c nude mice. When the xenograft tumours were allowed to grow for 10 days, the mice were randomly allocated into two groups, siNC and si*2R5C* group (*n* = 3 for each group), and then injected with 10 nmol/tumour/time siNC or si*2R5C* at the 1st and the 8th day. The mice were sacrificed and examined for tumour metastasis in the liver until the last injection week.

For tumour growth assay in vivo, the mice were randomly divided into four groups: siNC, si*2R5C*, HBx + siNC, and HBx + si*2R5C* (*n* = 4 for each group). HepG2 cells were transfected with pcDNA3.1‐HA or pcDNA3.1‐HA‐*HBX* plasmids by using Lipofectamine® 2000. Then the cells were selected with 200 μg/ml hygromycin B for 2 weeks. Exactly 5 × 10^6^ HBx‐expressing cells or control cells were suspended in cold phosphate‐buffered saline (PBS) with matrigel in a 1:1 ratio. The transfected cells were subcutaneously inoculated into the right flank of each mouse, and the tumours were allowed to grow for 10 days. Then, mice in the siNC and HBx + siNC groups were injected with 10 nmol/tumour siNC, and mice in the si*2R5C* and HBx + si*2R5C* groups were injected with 10 nmol/tumour si*2R5C* twice on the 11th and the 14th day (represents the 1st and the 4th day). All mice were sacrificed by cervical dislocation on the 17th day (represents the 7th day), and the xenograft tumours were removed.

Tumour width (*W*), tumour length (*L*), and body weights were measured. Tumour volume (*V*) was calculated with the formula: *V* = (*W* ^2^ × *L*)/2. Xenograft tumours and livers were collected and subjected to WB, HE, and IHC assays. All animal experiments were approved by the Experimental Animal Ethics Committee of Xiamen University.

### 2.6 HE and IHC analysis

The tumour tissue sections from HCC patients and BALB/c nude mice and serial sections of liver tissue from HBx‐Tg mice were fixed in 4% paraformaldehyde (pH 7.4) and embedded in paraffin. HE and IHC assays were performed as described previously.^19^ Briefly, the sections were rehydrated and stained with HE after dewaxing. The IHC sections were incubated with primary antibodies (anti‐HBx, anti‐AKT, anti‐MMP2, anti‐MMP9, and anti‐B56γ), and images were taken by an inverted microscope (Nikon, Tokyo, Japan).

### 2.7 Cell culture

HepG2, HepG2‐Tet‐ON‐HBx, HepG2.2.15, MHCC97H, and HEK‐293 T cell lines were maintained in our laboratory. HepG2‐Tet‐ON‐HBx cells, in which HBx expression can be induced with doxycycline (DOX)‐inducible promoter, were established and used as reported previously.^6^ HepG2.2.15 cells were derived from HCC cell line HepG2 with stable transfection of HBV genome expression. The HepG2, HepG2‐Tet‐ON‐HBx, and HepG2.2.15 cells were cultured in RPMI‐1640 medium (Gibco, California). MHCC97H and HEK‐293T cells were cultured in Dulbecco's Modified Eagle's medium (DMEM) (Gibco, California). Both media were supplemented with 10% foetal bovine serum (FBS) and 1% (v/v) penicillin–streptomycin. The cells were cultured at 37°C in a 5% CO_2_ humidified incubator (Thermo, California).

### 2.8 Targeting blockade of intracellular HBx with the recombinant anti‐HBx monoclonal antibody expression plasmid

The anti‐HBx monoclonal antibody (mAb) expression plasmid was constructed to block intracellular HBx expression.^20^ Briefly, to construct recombinant anti‐HBx mAb expression plasmid, the variable region gene of the cell clone (9D11) of anti‐HBx mAb was obtained from the hybridoma by reverse‐PCR using a mouse Ig primer set (Merck Millipore, Darmstadt, Germany). The coding sequences for the kappa chain and heavy chain of the anti‐HBx mAb were cloned into a pTT5 vector containing the constant region of mouse IgG1 and named pTT5‐anti‐HBx(9D11) plasmid after identification. The pTT5‐anti‐HBx(9D11) plasmid was transiently transfected into different HBx‐expression cells (HepG2.2.15 and HepG2‐Tet‐ON‐HBx cells) for 24 h to establish the blockade of intracellular HBx expression models, to verify the biological functions of anti‐HBx mAb specific targeting to block intracellular HBx.

### 2.9 Site‐directed mutagenesis of p‐AKT at Thr308 and Ser473

To prove the functional regulatory dephosphorylation of p‐AKT at Thr308 and Ser473, we simulated dephosphorylation (alanine mutation from serine or threonine, S or T to A) and the counterpart phosphorylation (aspartate mutation from serine or threonine, S or T to D) using recombinant expression plasmids. The specific site‐directed mutation method was used to convert the base in the coding sequence (CDS) by inverse PCR with KOD‐Plus Mutagenesis Kit (Toyobo, Osaka, Japan). As a CDS template, pcDNA3.1‐*AKT* was completely amplified with two primers in the opposite direction to generate base mutation from serine/threonine to alanine (S/T to A) or aspartate (S/T to D) at Thr308, or Ser473 of AKT (named as AKT^T308A^, AKT^S473A^, AKT^T308D^, and AKT^S473D^, respectively). Then, the PCR products were self‐ligated by T4 polynucleotide kinase. The primers are shown in Table S1. To construct the simulation of dephosphorylated or phosphorylated AKT expression, HepG2 cells were transfected with pcDNA‐3.1, pcDNA‐3.1‐AKT^WT^, ‐AKT^T308A^, ‐AKT^S473A^, ‐AKT^T308D^, or ‐AKT^S473D^ (1 μg/ml) using Lipofectamine® 2000 (Invitrogen).

### 2.10 Construction of stable B56γ‐knockdown and ‐overexpression cell lines

The specific method was mentioned in the previous experiment.^12^ For constructing the HepG2‐sh*2R5C* cells with B56γ‐knockdown expression, three different pLKO.1‐shRNA recombinant plasmids targeting various sites at 268, 417, and 1416 nt of *PPP2R5C* encoding sequence were transfected to HepG2 cells. For constructing the HepG2‐*2R5C* cells with B56γ‐overexpression, the *PPP2R5C* encoding sequence was incorporated into retroviral vector pBabe‐puro (pB) to construct the pBabe‐*2R5C* recombinant plasmid (pB‐*2R5C*). The established B56γ‐knockdown cell lines (HepG2‐sh*2R5C*) and B56γ‐overexpression cell lines (HepG2‐*2R5C*) were screened with 0.6 μg/ml puromycin. All the primers are shown in Table S2.

### 2.11 Establishment of genetic intervention cell models

To establish a transient HBx‐expression cell model, HepG2 cells were transfected with either pcDNA3.1 or pcDNA3.1‐*HBX* plasmids (1 μg/ml) using Lipofectamine® 2000 (Invitrogen).

For constructing an intracellular blockade of the HBx‐expressing cell model, HepG2 cells were transfected with either pTT5 or pTT5‐anti‐HBx(9D11) plasmid (1 μg/ml), which was without or with the production of anti‐HBx(9D11) mAb that targeted to intracellular HBx, using Lipofectamine® 2000 (Invitrogen).

For constructing a transient B56γ or AKT‐knockdown cell model, cells were transfected with 50 nM annealed double‐stranded small interfering RNA (siRNA) for *PPP2R5C* (si*2R5C*: 5′‐GGATTTGCCTTACCACTAA‐3′), *AKT* (si*AKT*: 5′‐CTCACCCAGTGACAACTCA‐3′) (Ribobio, Guangzhou, China) using Lipofectamine® 2000 according to the manufacturer's direction. siNC was used for siRNA negative control.

### 2.12 Quantitative real‐time polymerase chain reaction

Quantitative real‐time polymerase chain reaction (qRT‐PCR) analysis was performed as described previously.^12^ The relative expression of *PPP2R5C* was determined by comparing the target genes' threshold cycle (Ct) using the 2^−ΔΔCt^ method. The following primers were used for detection: *PPP2R5C* forward: 5′‐CAAAGCCAATCCCCAGTAC‐3′, reverse: 5′‐TCGGATCTTTCTGTGCCTGA‐3′. *ACTB* forward: 5′‐CACCAGGGCGTGATGGT‐3′, reverse: 5′‐CTCAAACATGATCTGGGTCAT‐3′.

### 2.13 WB analysis

Cells, liver tissues, and xenograft tumour tissues were lysed in sodium dodecyl sulphate (SDS) buffer with 1% protease and phosphatase inhibitor cocktails. The indicated protein bands were incubated overnight at 4°C with primary antibodies of the target proteins: anti‐HBx (Abcam, Massachusetts, 1:2000), anti‐MMP2, and anti‐MMP9 (Ruiying Biological, Suzhou, China, 1:1000), anti‐GAPDH, anti‐E‐cadherin, and anti‐vimentin (Beyotime, Shanghai, China, 1:1000), anti‐AKT, anti‐p‐AKT^Thr308^, and anti‐p‐AKT^Ser473^ (Cell Signalling Technology, Massachusetts, 1:1000), anti‐B56γ (Thermo, Massachusetts, 1:1000). It was worth noting that the sample was directly incubated with the secondary antibody without first incubating the primary antibody when the cell was transfected with anti‐HBx mAb expression plasmid, pTT5‐anti‐HBx(9D11). Signals were visualized using the Azure Biosystems (Beijing, China).

### 2.14 Immunofluorescence analysis

For immunofluorescence (IF) staining, serial tissue sections and cell‐mounted slides were permeabilized with 0.5% Triton X‐100 on ice for 5 min, then blocked by 1% BSA. After incubating with the primary antibodies (anti‐HBx, anti‐B56γ, anti‐MMP2, anti‐MMP9, anti‐p‐AKT^Thr308^, and anti‐p‐AKT^Ser473^) at 4°C overnight, the samples were incubated with goat anti‐mouse/rabbit IgG fluorescent secondary antibody for 1 h at room temperature, and then counterstained with DAPI to visualize the cell nuclei. Finally, stained sections were visualized using a confocal microscope (Zeiss LSM 880, Wetzlar, Germany). Fluorescence curves of profile lines were generated using Zen 2010 software. It was worth noting that the sample was directly incubated with the secondary antibody containing fluorescent probes (647 nm) without first incubating the primary antibody when the cell was transfected with pTT5‐anti‐HBx(9D11).

### 2.15 Wound healing assay

According to a previous study, cell migration was determined using the wound healing assay according to the previous study.^21^ Each well was photographed using a microscope (Nikon, Tokyo, Japan) to estimate the relative cell migration. The unhealed distance was measured using Image J software.

### 2.16 Transwell invasion assay

Cells were treated as described above for 24 h before being pipetted into the upper chamber of the transwell. Transwell invasion assay was performed according to the previous study.^21^ We randomly chose five fields of vision to count cells under a digital camera (Canon, Tokyo, Japan). The number of invaded cells was calculated.

### 2.17 Statistical analysis

Statistical analyses were performed using the Statistical Package for Social Sciences (SPSS) version 20.0 (SPSS, Chicago, Illinois). All data are expressed as mean ± standard deviation (SD) of at least three independent experiments. Statistical significance was determined using the Student's *t*‐test and one‐way analysis of variance (ANOVA). Pearson's correlation analysis was performed for the correlation between variables. A *P‐*value of <0.05 was considered statistically significant.

## 3 RESULTS

### 3.1 Cell migration and the AKT pathway were associated with hepatocarcinogenesis in bioinformatics analysis of HBV‐infected liver and HCC tissue

**FIGURE 1:**
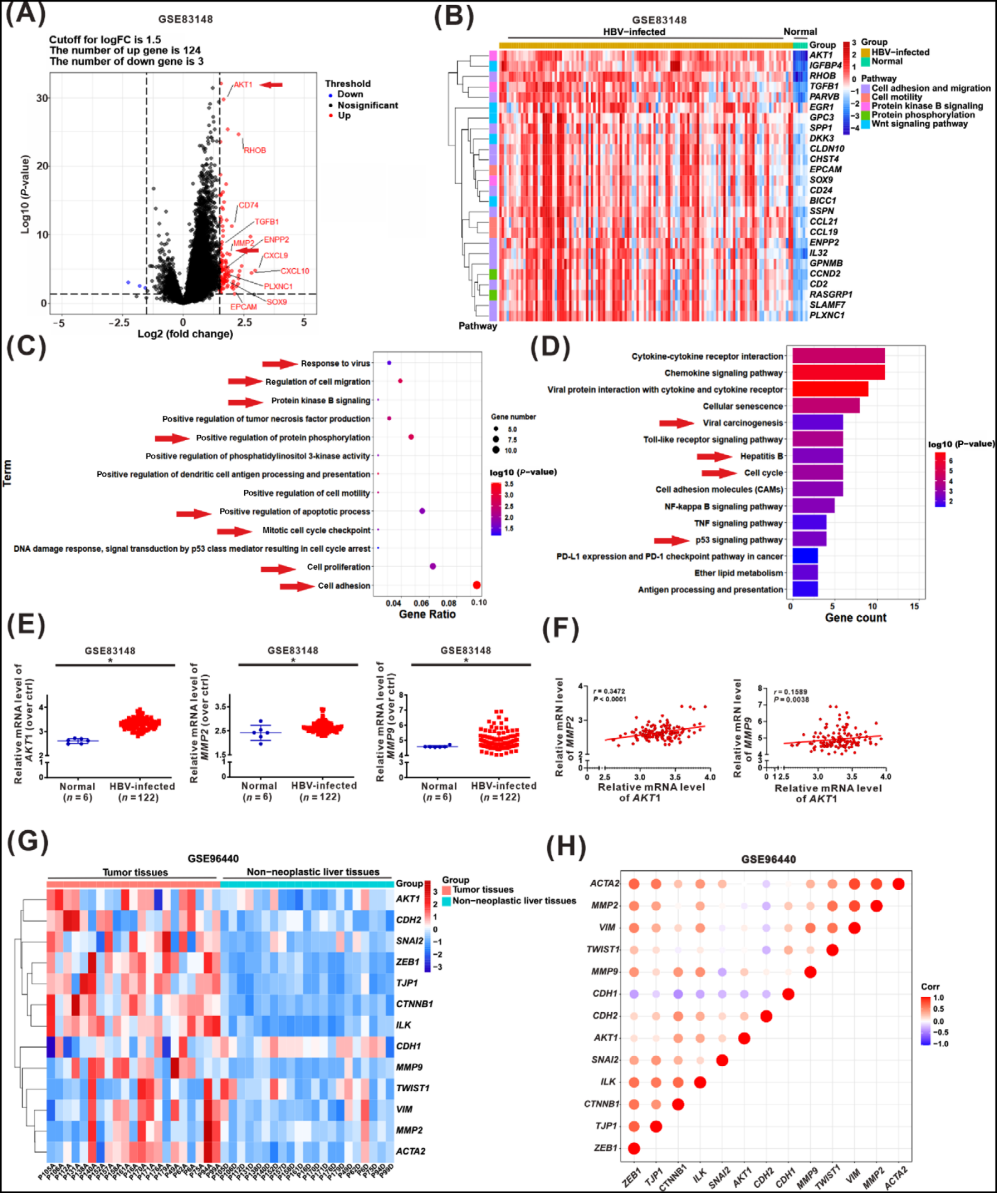
Cell migration and the AKT pathway were associated with hepatocarcinogenesis in bioinformatics analysis of HBV‐infected liver and HCC tissue. (A–F) GEO database (accession no.: GSE83148) of gene expression in HBV‐infected hepatitis samples. (A) The volcano plot was displayed for DEGs analysis. Red plots and blue plots (Log_2_FC ≥1.5 or ≤−1.5, and *P* < 0.05) represented the up‐ or down‐regulated genes in the HBV‐infected liver tissues compared with normal liver tissues, respectively. The cell malignant transformation‐related genes were tagged with red arrows. (B) The heatmap of DEGs in the HBV‐infected tissues (*n* = 122) and normal liver tissues (*n* = 6). (C) GO enrichment was displayed for biological process (BP) analysis of DEGs. (D) KEGG enrichment was displayed for analysis of DEGs. (E) The relative mRNA levels of *AKT1*, *MMP2*, and *MMP9* in normal liver tissues and HBV‐infected liver tissues were shown; the ‘*n*’ represented the number of samples. (F) Pearson's correlation analysis was used for the relationship between *AKT1* and *MMP2*, *AKT1* and *MMP9* expression levels. *P < 0.05. (G–H) GEO database (accession no.: GSE94660) of gene expression in HBV‐related HCC samples. (G) The heatmap of DEGs in the HBV‐related HCC tumour tissues (*n* = 21) and the paired non‐neoplastic liver tissues (*n* = 21). (H) Pearson's correlation analysis was used for the relationship between mRNA levels of oncogenic phenotype‐related genes.

The GEO database (accession no.: GSE83148) of HBV‐infected hepatitis samples was screened and underwent DEGs analysis to examine the gene expression landscape of HBV‐related hepatocarcinogenesis. We found 124 genes that were upregulated and 3 genes that were downregulated in the HBV‐infected liver tissues (*n* = 122) relative to normal liver tissues (*n* = 6) (Figure 1A). The levels of *AKT1* (*AKT* isoform) and genes related to malignant cell transformation were significantly upregulated in the HBV‐infected liver tissues (Figure 1B). We used GO enrichment analysis for biological processes (BP) to identify the genes significantly affected by HBV infection to determine whether HBV infection was associated with cell migration. As a result, we found that ‘response to virus’, ‘cell migration’, ‘protein kinase B signalling’, and ‘regulation of protein phosphorylation’ terms were significantly enriched in the HBV‐infected liver tissues (Figure 1C). KEGG pathway enrichment analysis further demonstrated that ‘viral carcinogenesis’, ‘Hepatitis B’, ‘cell cycle’, and ‘p53 signalling pathway’ terms were significantly enriched in the HBV‐infected liver tissues (Figure 1D), which was consistent with the result of our previous study, which showed that HBx could regulate cell cycle arrest and apoptosis through regulating p‐p53^Thr55^ dephosphorylation by B56γ.^10^ Moreover, in HBV‐infected liver samples, the expression of *AKT1*, *MMP2*, and *MMP9* were upregulated compared with that observed in normal liver samples (Figure 1E). Upon further exploration, we identified a positive correlation between the gene expression of *AKT1* and *MMP2* and that of *AKT1* and *MMP9* (Figure 1F).

We used another GEO database (accession no.: GSE94660) to screen and perform DEGs analysis of the relative gene expression in HBV‐positive infected tumour tissues and their paired non‐neoplastic liver tissues. The relative expressions of *AKT1*, *MMP2*, and *MMP9* genes were higher in tumour tissues than in non‐neoplastic liver tissues (Figure 1G). Moreover, there was a positive correlation between *AKT1* and *MMP2* and *AKT1* and *MMP9* expressions in HCC samples (Figure 1H). Furthermore, the Cancer Genome Atlas (TCGA) dataset with a higher number of HCC patients was adopted additionally. The analysis revealed that the relative expressions of *AKT1*, *MMP2*, and *MMP9* genes were increased in HCC tumour tissues (*n* = 373) compared with the normal samples (*n* = 50; Figure S1A). There was a positive correlation between *AKT1* and *MMP2* and *AKT1* and *MMP9* expression in HCC samples (Figure S1B). Moreover, the trend relationship analysis revealed that the relative expressions of *AKT1* were upregulated in HCC tumour tissues compared with the normal samples (Figure S1C). These results demonstrated that HBV infection was positively correlated with cell migration and AKT signalling, suggesting a potential regulatory mechanism of AKT signalling involved in HBx‐associated hepatocarcinogenesis via regulating cell migration.

### 3.2 Expression of HBx promoted the phosphorylation of AKT signalling to activate the migration phenotype in the livers of HBx‐Tg mice

HBx is encoded by the HBV genome and plays an important role in HBV infection and the development of HBV‐associated HCC. HBV infection was associated with cell migration and protein kinase B signalling (Figure 1C). However, the exact role of HBx‐mediated signal regulation (such as PTM of target molecule) in cell migration was unclear. The effects of HBx‐expression on liver regeneration have been demonstrated in two independent lines of HBx transgenic (HBx‐Tg) mice, both of which developed HCC at 14–16 months of age.^22^ In our previous study, we used 12‐month‐old HBx‐Tg mice with continuous HBx‐expression to induce liver carcinogenesis and revealed the bioeffect of HBx in HBV‐associated apoptosis and hepatic injury.^12^ We further wish to elucidate whether HBx promotes cell migration and signalling transduction of liver carcinogenesis in long‐term expression HBx‐Tg mice.

**FIGURE 2:**
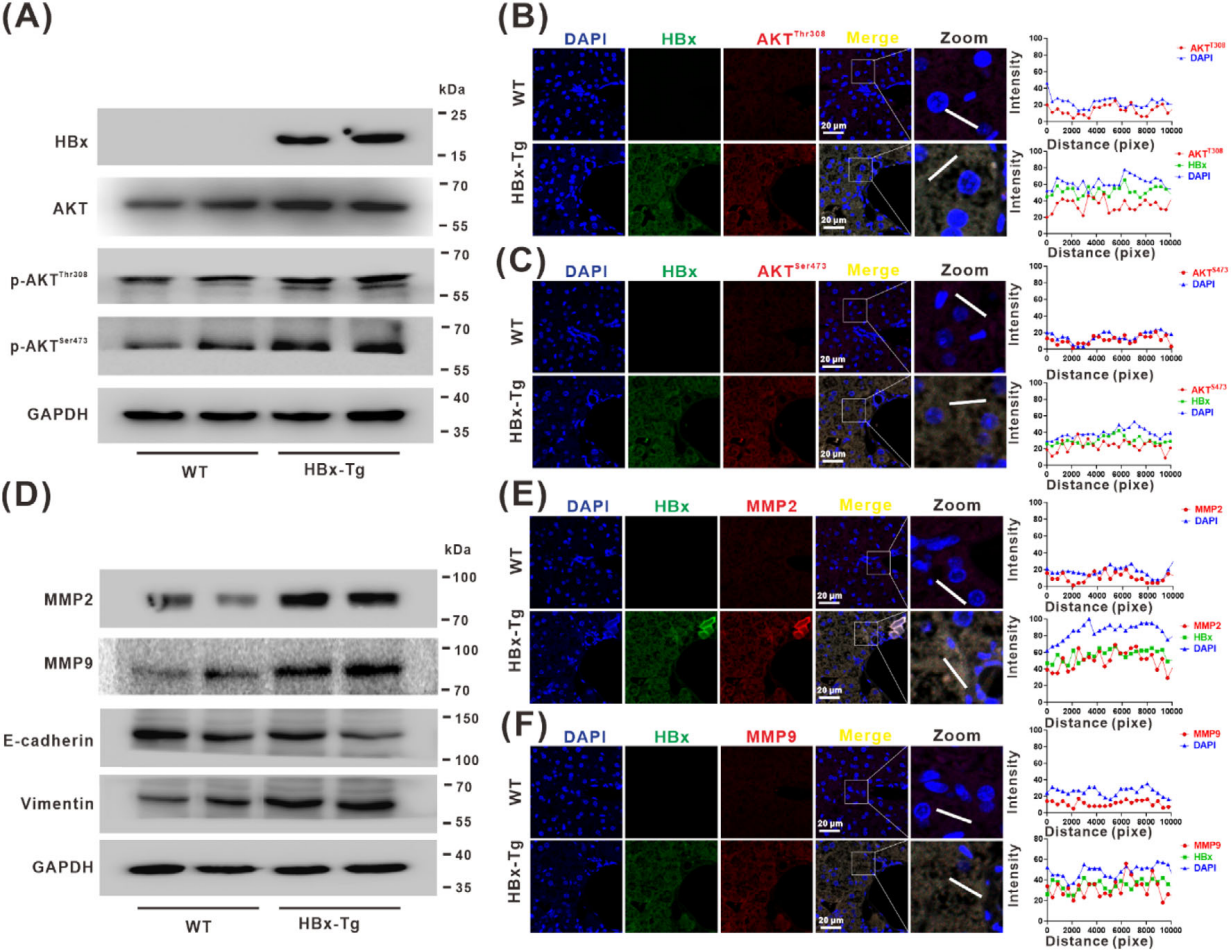
Expression of HBx promoted the phosphorylation of AKT signalling to activate the migration phenotype in the livers of HBx‐Tg mice. The 12‐month‐old HBx‐Tg mice (*n* = 3) with continuous long‐term HBx expression and their corresponding wild‐type (WT) mice (*n* = 3) were used. (A) The protein levels of HBx, AKT, p‐AKT^Thr308^, and p‐AKT^Ser473^ were detected by Western blot. (B,C) Representative images for evaluating the expression of HBx (green), p‐AKT^Thr308^ (red), or p‐AKT^Ser473^ (red), and the colocalization (yellow) between HBx and p‐AKT^Thr308^ or p‐AKT^Ser473^ (Left). Profile line of fluorescence curves were generated using Zen 2010 software (Right). (D) The protein levels of MMP2, MMP9, E‐cadherin, and vimentin were detected by Western blot. (E, F) Representative images for evaluating the expression of HBx (green), MMP2 (red), or MMP9 (red), and the colocalization (yellow) between HBx and MMP2 or MMP9 (Left). Fluorescence curves of profile lines were generated using Zen 2010 software (Right). DAPI (Blue) was for nucleus staining. Scale bars, 20 μm.

Our results demonstrated that the protein levels of phosphorylated AKT at Thr308 (p‐AKT^Thr308^) and Ser473 (p‐AKT^Ser473^), which are essential for AKT pathway activation, were upregulated with increasing the expression of HBx in long‐term (12‐month‐old) HBx‐Tg mice (Figure 2A). Immunofluorescence staining of serial sections of liver tissues showed that the levels of p‐AKT^Thr308^ and p‐AKT^Ser473^ were increased and highly expressed in HBx‐Tg mice (Figure 2B,C). Matrix metalloproteinases (MMPs), including MMP2 and MMP9, have been previously reported to promote cell migration and tumour metastasis.^23^ Meanwhile, the levels of MMP2 and MMP9 (Figure 2D) and their distribution in liver cells upregulated with the increased expression of HBx (Figure 2E,F). Concerning the phenotypic markers of metastasis in hepatocarcinogenesis, the level of EMT‐related protein E‐cadherin was downregulated in long‐term HBx‐Tg mice, while vimentin was upregulated (Figure 2D). These results indicated that HBx‐expression can promote the cell migratory phenotype during hepatocarcinogenesis and that the phosphorylation of AKT and subsequent signalling activation may be involved in this process.

### 3.3 Phosphorylation of AKT^Thr308^ ^/Ser473^ regulated the migration and invasion of HBx‐expressing cells

**FIGURE 3:**
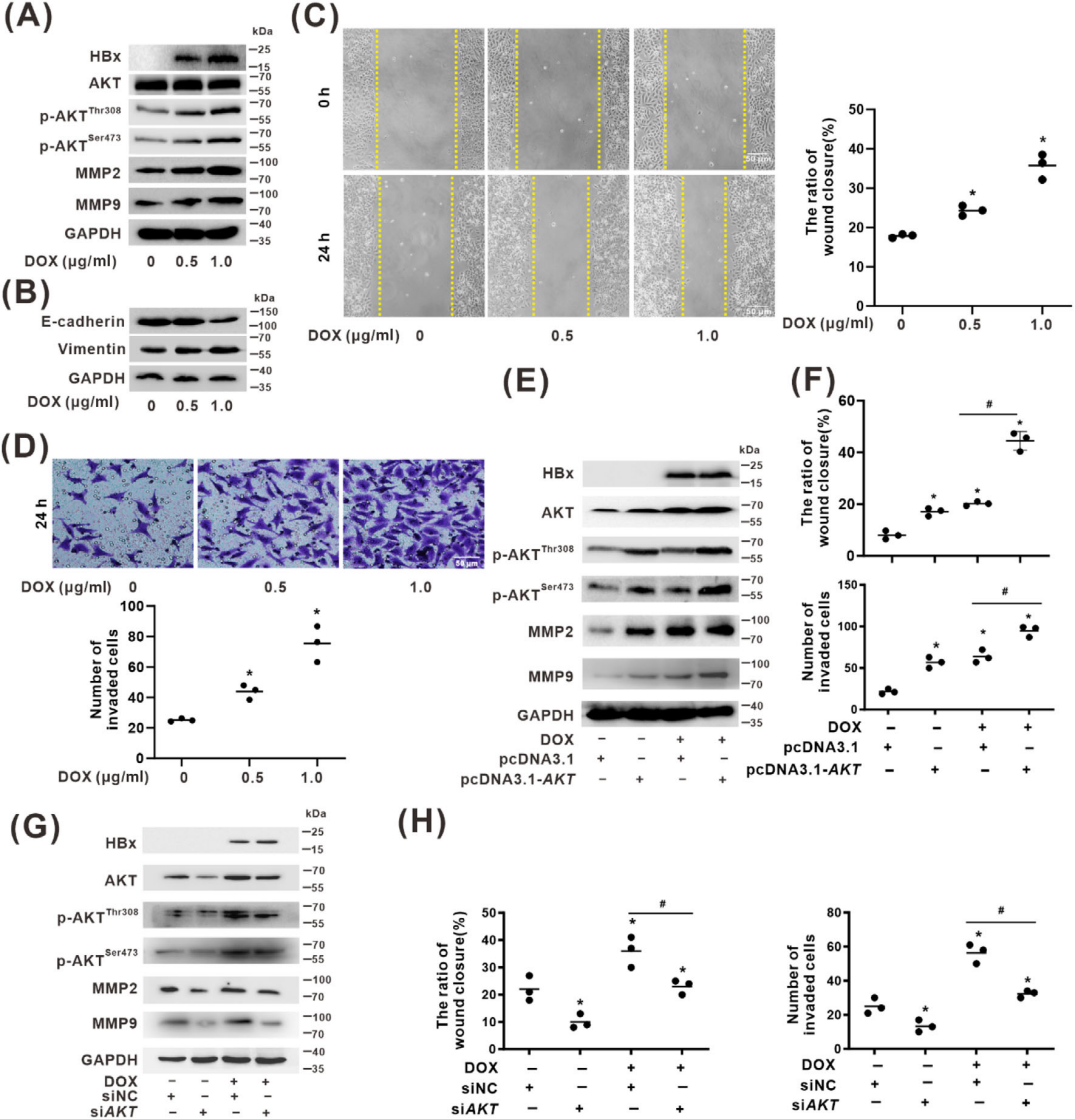
Phosphorylation of AKT^Thr308/Ser473^ regulated the migration and invasion of HBx‐expressing cells. (A–D) HepG2‐Tet‐ON‐HBx cells treated with DOX were used as a model in which HBx‐expression can be induced in a dose‐dependent manner by different concentrations of DOX (0.5, 1 μg/ml) for 24 h. (A) The protein levels of HBx, AKT, p‐AKT^Thr308^, p‐AKT^Ser473^, MMP2, and MMP9 were detected by Western blot. (B) The protein levels of E‐cadherin and vimentin were detected by Western blot. (C) A wound‐healing assay was conducted to evaluate cell migration ability. Representative images were shown (Left). The relative quantification ratio of wound closure in each field of view is shown in the scatter plot (Right). Scale bar, 50 μm. *P < 0.05, compared with the control group. (D) The transwell invasion assay was conducted to evaluate HCC‐related invasion. Representative images were shown (Upper). The relative quantification of the number of invaded cells in each field of view is shown in the scatter plot (Lower). Scale bar, 50 μm. *P < 0.05, compared with the control group. (E,F) AKT overexpression model was established in HBx‐expressing HCC cells, in which HepG2‐Tet‐ON‐HBx cells were treated with DOX (1 μg/ml) and transfected with pcDNA3.1 or pcDNA3.1‐*AKT* for 24 h. (E) The protein levels of HBx, AKT, p‐AKT^Thr308^, p‐AKT^Ser473^, MMP2, and MMP9 were detected by Western blot. (F) The scratch width and the number of invaded cells were evaluated by wound healing and transwell invasion assays. The relative quantification ratio of wound closure (Upper) and the number of invaded cells (Lower) in each field of view are shown in the scatter plots. *P < 0.05, compared with the pcDNA3.1 group. ^#^ *P* < 0.05, compared with the HBx‐expressing group treated with DOX and transfected with pcDNA3.1. (G,H) Small interfering RNA targeting to *AKT* gene (si*AKT*) was used to knock down AKT expression in HBx‐expressing cells, which HepG2‐Tet‐ON‐HBx cells were treated with DOX (1 μg/ml) and transfected with siNC or si*AKT* for 24 h. siNC was served as the negative control (NC). (G) The protein levels of HBx, AKT, p‐AKT^Thr308^, p‐AKT^Ser473^, MMP2, and MMP9 were detected by Western blot. (H) The scratch width and the number of invaded cells were evaluated by wound healing and transwell invasion assays. The relative quantification ratio of wound closure (Left) and the number of invaded cells (Right) in each field of view are shown in the scatter plots. *P < 0.05, compared with the siNC group. ^#^ *P* < 0.05, compared with the HBx‐expressing group treated with DOX and transfected with siNC.

We performed a wound‐healing assay and transwell invasion assay in vitro to determine the promoting effects of HBx on the regulation of cellular migration and invasion of HCC. We found inducible higher HBx‐expression and the increased levels of p‐AKT^Thr308^ and p‐AKT^Ser473^ in doxycycline (DOX)‐treated HepG2‐Tet‐ON‐HBx cells in a dose‐dependent manner (Figure 3A). It has been reported that p‐AKT activation is crucial in HCC progression,^24^, ^25^ and MMP2 and MMP9 are involved in tumour invasion and metastasis.^26^ As we found, the protein levels of MMP2 and MMP9 were upregulated with the increased expression of HBx in HepG2‐Tet‐ON‐HBx cells (Figure 3A). The protein level of E‐cadherin was downregulated in DOX‐treated HepG2‐Tet‐ON‐HBx cells in a dose‐dependent manner, while vimentin was upregulated (Figure 3B).

Moreover, in HepG2‐Tet‐ON‐HBx cells, HBx‐expression promoted cell migration phenotype, which showed a shorter distance of scratch width than the control group (Figure 3C), and increased invasion as evidenced by an increase in the number of invaded cells compared with the control group (Figure 3D) in an HBx‐dependent manner. Next, to further confirm the regulatory effect of AKT signalling on HBx‐induced cell migration and invasion, gain or loss of function assays were conducted in AKT overexpression and knockdown cell models with HBx‐expressing cells. It was found that the levels of p‐AKT^Thr308^ and p‐AKT^Ser473^, and the expression of MMP2 and MMP9, were upregulated in the AKT‐overexpression HCC cells with HBx‐expression (Figure 3E). Consistently, the cellular migration ability was enhanced, and the number of invaded cells was augmented in the AKT‐overexpression cells, with the greatest effects observed in HBx‐expressing cells (Figures 3F and S2A). In addition, the increased levels of p‐AKT^Thr308^ and p‐AKT^Ser473^ and the upregulated expression of MMP2 and MMP9 by HBx‐expression were inhibited in the AKT‐knockdown cells (Figure 3G). The promotion effects of HBx on cell migration and invasion were counteracted when AKT expression was inhibited by specifically suppressing *AKT* transcripts using si*AKT* (Figures 3H and S2B). These results suggested that HBx‐expression can promote migration and invasion via regulating the phosphorylation of p‐AKT^Thr308/Ser473^ in HCC cells.

### 3.4 Dephosphorylation of p‐AKT^Thr308^ ^/Ser473^ was involved in the migration and invasion of HCC cells, and B56γ correlated with HBx expression in HBV‐infected HCC tissues

**FIGURE 4:**
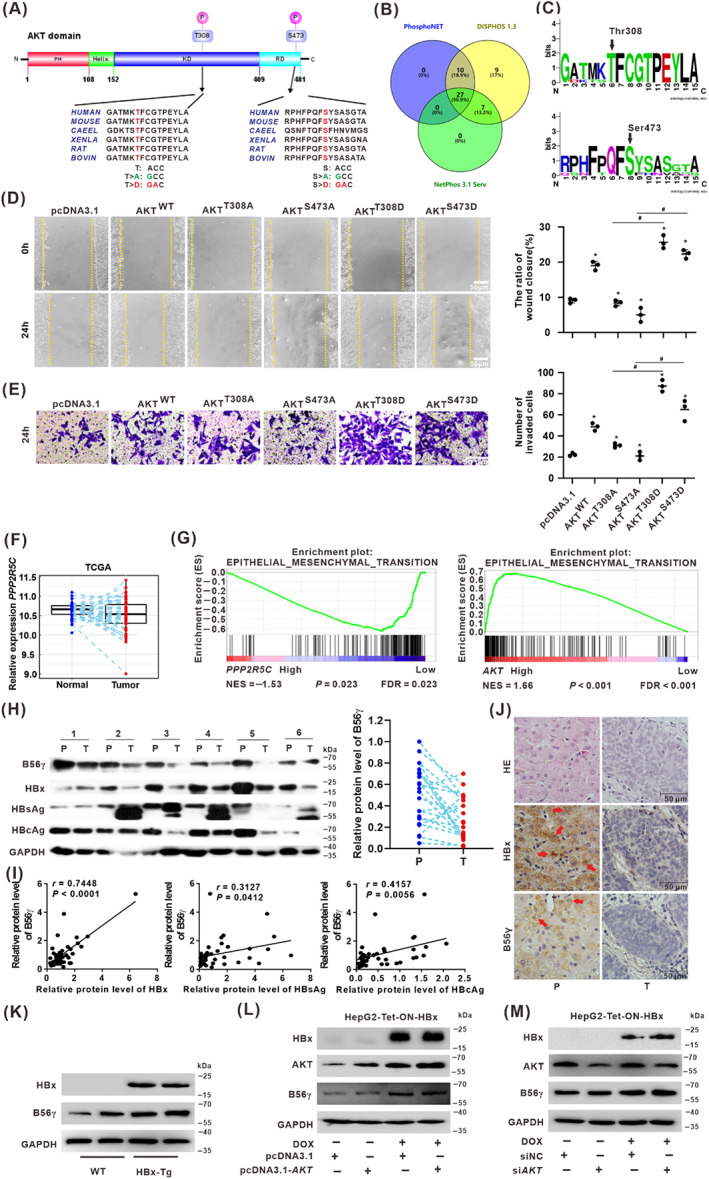
Dephosphorylation of p‐AKT^Thr308/Ser473^ was involved in the migration and invasion of HCC cells, and B56γ correlated with HBx expression in HBV‐infected HCC tissues. (A) The schematic structure of the indicated AKT domain was illustrated. The conserved amino acid sequence of serine/threonine phosphorylation sites Thr308 and Ser473 was shown among different species, while P shows the potential phosphorylation. Based on the encoding bases of these two sites, site‐directed mutation expression plasmids (pcDNA‐3.1) mimics dephosphorylation (T > A, S > A) or phosphorylation (T > D, S > D) were constructed. (B) Venn diagram of AKT phosphorylation sites predicted by PhosphoNET (blue), NetPhos 3.1 Server (green), and DISPHOS1.3 (yellow) website. (C) The conserved Thr308 (Upper) and Ser473 (Lower) of AKT phosphorylation sites were shown (generated at http://weblogo.berkeley.edu/logo.cgi). (D, E) HepG2 cells were transfected with pcDNA‐3.1, pcDNA‐3.1‐AKT^WT^, ‐AKT^T308A^, ‐AKT^S473A^, ‐AKT^T308D^, or ‐AKT^S473D^ for 24 h to establish the simulation of dephosphorylated or phosphorylated AKT expression. Wound healing assay (D) and transwell invasion assay (E) were conducted to evaluate the migration ability and HCC‐related invasion of HepG2 cells with the mimics expression of dephosphorylated or phosphorylated AKT (Left). The relative quantification ratio of wound closure and the number of invaded cells in each field of view are shown in the scatter plots (Right). Scale bar, 50 μm. *P < 0.05, compared with the pcDNA‐3.1‐AKT^WT^ group cells, ^#^ *P* < 0.05, compared with the pcDNA‐3.1‐AKT^T308A^ or ‐AKT^S473A^ group cells. (F) *PPP2R5C* was significantly upregulated in HCC tumour tissues based on the trend relationship analysis of the TCGA datasets. (G) GSEA indicated the significant correlations between the expression of *PPP2R5C* (encoding B56γ) (Left) or *AKT* (encoding AKT) (Right) and epithelial‐mesenchymal transition (EMT) signatures. (H–J) Twenty‐two paired peritumor (P) and tumour (T) specimens from HCC patients were detected. (H) Expressions of B56γ, HBx, HBsAg, and HBcAg proteins in representative six paired specimens were detected by Western blot (Left). The trend relationship analysis for B56γ expression in the 22 pairs of peritumor (P) and tumour (T) tissues were shown (Right). (I) Pearson's correlative regression analysis for the expression of B56γ with HBx, HBsAg, or HBcAg, respectively. (J) Representative images of HE and IHC staining of HBx and B56γ in peritumor and tumour tissues. Red arrows indicated HBx and B56γ expressions. Scale bars: 50 μm. (K) The levels of HBx and B56γ expression in livers were detected in the 12‐month‐old HBx‐Tg mice and the corresponding WT mice. (L, M) AKT overexpression (L) and knockdown AKT (M) model was established in HBx‐expressing HCC cells, which HepG2‐Tet‐ON‐HBx cells were treated with DOX (1 μg/ml) and transfected with pcDNA3.1 or pcDNA3.1‐*AKT* for 24 h. The levels of HBx and B56γ were evaluated by Western blot.

The above experiments demonstrated that p‐AKT^Thr308/Ser473^ was necessary for HBx‐induced migration and invasion. Therefore, to investigate the role of regulatory dephosphorylation of p‐AKT^Thr308/Ser473^ in hepatocarcinogenesis, we performed a functional analysis of p‐AKT^Thr308/Ser473^ dephosphorylation modification on HCC‐associated cell migration and invasion phenotypes. We blasted the amino acid sequence of AKT in different species for conservation analysis. The results demonstrated that AKT contains multiple functional domains, including the pleckstrin homology (PH) domain, HELIX domain, kinase domain (KD), and regulation domain (RD; Figure 4A). PhosphoNET, NetPhos 3.1 server, and DISPHOS1.3 websites were used to predict the phosphorylation site of AKT PTM. Twenty‐seven phosphorylation sites were screened with the common prediction (Figure 4B), including the conserved Thr308 and Ser473 of AKT (Figure 4C). Thr308 is on the KD domain, and Ser473 is on the RD domain, where the amino acid sequences of both sites are conserved among different species (Figure 4A).

We constructed the recombinant expression plasmids for the mimics phosphorylation or dephosphorylation of AKT at Thr308 and Ser473 to further verify the functional dephosphorylated regulating p‐AKT^Thr308/Ser473^ (Figure 4A). In vitro, the wound healing assay showed that the migration ability of AKT^T308A^ and AKT^S473A^ expression cells was significantly decreased compared with the AKT^WT^ group cells, while the migration ability was significantly increased in AKT^T308D^ and AKT^S473D^ expression cells compared with the AKT^WT^ group, AKT^T308A^, and AKT^S473A^ group cells (Figure 4D). A similar effect was confirmed by the results of the transwell invasion assay, which demonstrated a significant decrease in HCC‐associated invasion of AKT^T308A^ and AKT^S473A^ expression cells compared with AKT^WT^ group cells, while the invasion ability was significantly increased in AKT^T308D^ and AKT^S473D^ expression cells compared with AKT^T308A^ and AKT^S473A^ group cells (Figure 4E). These results demonstrated that dephosphorylation of p‐AKT^Thr308/Ser473^ inhibited the migration and invasion of HCC cells.

Our previous study found that HBx expression in hepatocytes upregulated the expression of B56γ (encoding by the *PPP2R5C* gene) via endoplasmic reticulum (ER) stress to induce cell cycle arrest and apoptosis.^12^ To test whether B56γ expression was correlated with HCC progression, we analysed the TCGA dataset from 50 paired HCC and their corresponding normal samples. The analysis revealed that the relative expressions of *PPP2R5C* were downregulated in HCC tumour tissues compared with the normal tissues (Figure 4F). To explore the relationship between B56γ and AKT in hepatocarcinogenesis, we used GSEA to explore the significant signalling pathways regulated by *PPP2R5C* and *AKT* genes between low and high expression groups. The EMT signalling pathway was mainly focused on in the current study. It was shown that the normalized enrichment scores (NES) for the EMT signals were −1.53 and 1.66 (*P* < 0.05) for *PPP2R5C* and *AKT* genes, respectively, which suggested that the expression of *PPP2R5C* was negatively and *AKT* was positively correlated with EMT (Figure 4G). Furthermore, to investigate the potential association between B56γ and HBV‐related hepatocarcinogenesis, we collected 22 pairs of HBV‐related HCC tumours and adjacent peritumor tissues to analyse the levels of HBV proteins, including HBsAg, hepatitis B core antigen (HBcAg), HBx, and B56γ expression. It was shown that the expression of the B56γ subunit was significantly downregulated in tumour (T) tissues compared with the corresponding peritumor (P) tissues (Figures 4H and S3). As shown in Figure 4I, Pearson's correlation regression analysis showed that the expression of B56γ and HBx displayed a positive correlation (*r* = 0.7448, *P* < 0.0001) in the 22 paired specimens, while that of HBsAg and HBcAg showed a weaker correlation with the level of B56γ (*r* = 0.3127 and *r* = 0.4157, respectively). These data demonstrated that in patients with HCC, B56γ positively correlated with HBx‐expression and was considerably higher correlation than that observed with HBcAg or HBsAg. Representative IHC images showed the co‐overexpression of HBx and B56γ in both peritumor and tumour tissues, with higher levels observed in the peritumor than in the tumour tissues (Figure 4J).

Moreover, the levels of B56γ expression in the livers were significantly higher in the 12‐month‐old HBx‐Tg mice with HBx continuous long‐term expression than in the corresponding wild‐type (WT) mice in vivo (Figure 4K). In addition, the upregulated B56γ expression induced by HBx‐expression in HepG2‐Tet‐ON‐HBx cells remained at higher levels in AKT‐overexpression pcDNA3.1‐*AKT* group cells or AKT‐knockdown si*AKT* group cells than in the corresponding control group cells in vitro (Figure 4L,M). Collectively, B56γ might be activated in HBV‐related hepatocarcinogenesis but inactivated and downregulated in HCC tumour tissue. Moreover, B56γ induced by HBx‐expression might be involved in the upstream regulation of AKT signalling. Regulation of B56γ by targeting *PPP2R5C* expression in HBV‐ and HBx‐associated hepatocarcinogenesis should be further investigated.

### 3.5 Inhibition of B56γ promoted the migration and invasion of HBV‐related HCC cells in vitro and in vivo

**FIGURE 5:**
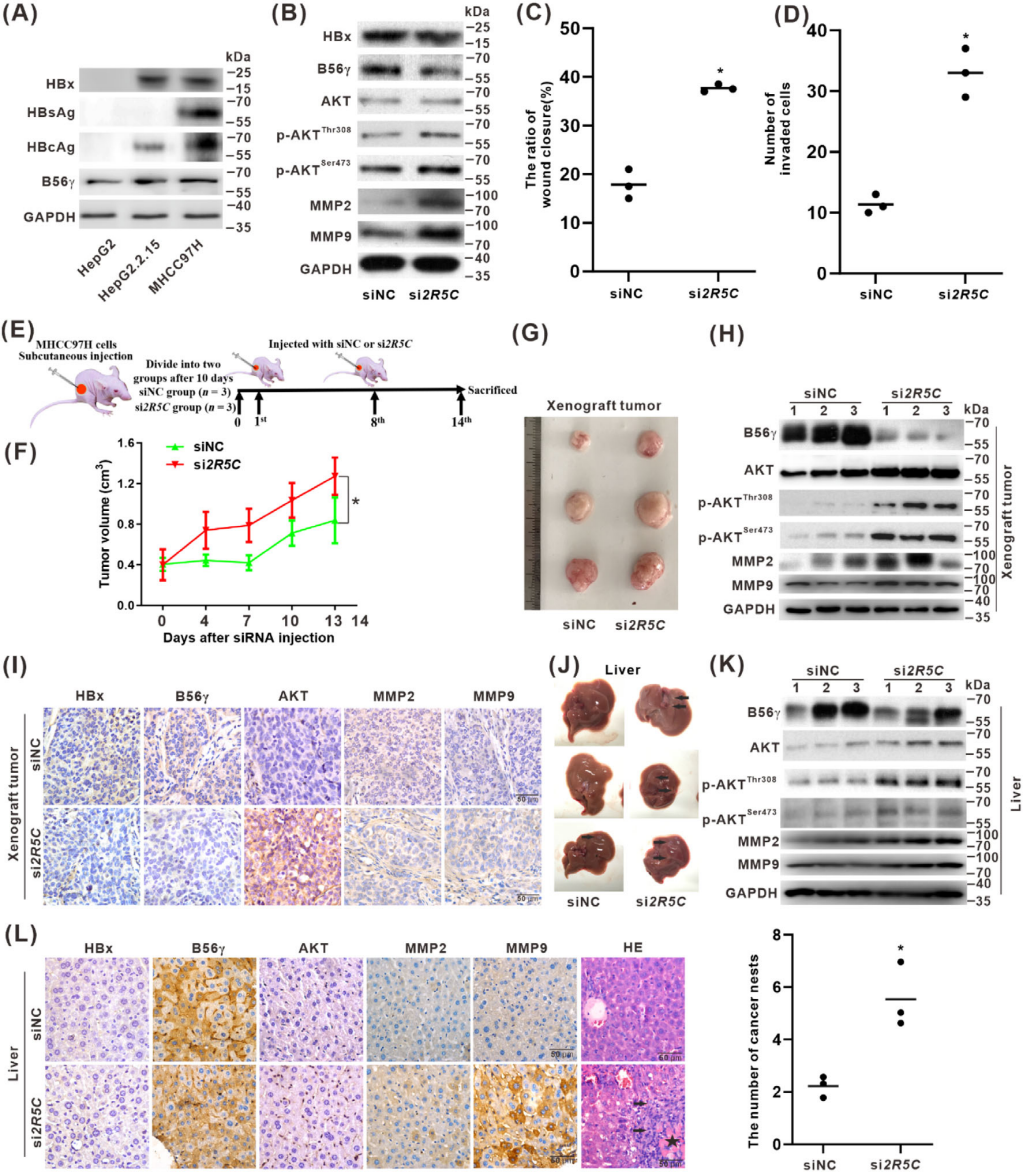
Inhibition of B56γ promoted the migration and invasion of HBV‐related HCC cells in vitro and in vivo. (A) The expressions of HBV proteins and B56γ were detected by Western blot in MHCC97H cells, HepG2 cells, and HepG2.2.15 cells. (B–D) MHCC97H cells were transfected with si*2R5C* for 24 h to knock down B56γ, while siNC served as negative control (NC). (B) The levels of B56γ, AKT, p‐AKT^Thr308^, p‐AKT^Ser473^, MMP2, and MMP9 were detected by Western blot. (C,D) A wound‐healing assay (C) and the transwell invasion assay (D) were conducted to evaluate cell migration and invasion ability. The relative quantification ratio of wound closure and the number of invaded cells in each field of view is shown in the scatter plot, respectively. *P < 0.05, compared with the siNC control group. (E–L) MHCC97H cells were subcutaneously inoculated into BALB/c nude mice. The mice were randomly allocated to two groups until the xenograft tumours grew to 0.4–0.5 cm^3^. Si*2R5C* group mice (*n* = 3), with the knockdown of B56γ, were tail‐vein injected with si*2R5C* twice on the 1st and the 8th day, while siNC group mice (*n* = 3) were served as negative control (NC). On the 14th day, mice were sacrificed, and examined the xenograft tumours. Xenograft tumours and livers were collected and subjected to analyses. (E) Schematic diagram of experimental design for B56γ targeting intervention in xenograft tumours of BALB/c nude mice. (F) The growth curves of xenograft tumours formed by MHCC97H cells were injected with si*2R5C* or siNC. *P < 0.05, compared with the siNC group. (G) Excised xenograft tumours were photographed after mice were sacrificed. (H) The expression of B56γ, AKT, p‐AKT^Thr308^, p‐AKT^Ser473^, MMP2, and MMP9 in the xenograft tumours were detected by Western blot. (I) Representative IHC images for the expression of HBx, B56γ, AKT, MMP2, and MMP9 in the xenograft tumours. Scale bar, 50 μm. (J) Excised livers from xenograft tumour‐bearing mice were photographed, and arrows indicated the HCC migrated nodules in the liver tissues. (K) The expression of B56γ, AKT, p‐AKT^Thr308^, p‐AKT^Ser473^, MMP2, and MMP9 in livers were detected by Western blot. (L) Representative IHC images of HBx, B56γ, AKT, MMP2, and MMP9 expression in livers. HE staining showed the cancer nests at the migrated nodules in livers from xenograft tumour‐bearing mice (Left), while arrows indicated HCC migrated nodules and a black asterisk indicated angiogenesis in migrated tumours in the si*2R5C* group. Scale bar, 50 μm. The number of cancer nests in each field of view was quantified in the scatter plot (Right). *P < 0.05, compared with the siNC control group.

We used MHCC97H cells, HBV genome‐integrated, and highly metastatic HCC cells,^27^ to further prove the regulatory role of B56γ in HBx‐associated migration and metastasis. It was found that the expression of B56γ increased in MHCC97H cells according to HBx expression (Figure 5A). The siRNA targeting *PPP2R5C* (si*2R5C*) was used to knock down B56γ in MHCC97H cells. In vitro, after si*2R5C* treatment, the expression of B56γ decreased, the levels of p‐AKT^Thr308/Ser473^ were upregulated, and the levels of MMP2 and MMP9 were increased (Figure 5B). As shown in the scratch test, the migration and the invasion of MHCC97H cells increased with si*2R5C* treatment (Figures 5C,D and S4A,B). Moreover, in vivo test (Figure 5E), knockdown of B56γ enhanced the growth and size of MHCC97H xenograft tumours in the si*2R5C* injection group (Figure 5F,G). In the HBx‐expressing MHCC97H xenograft tumour tissues, si*2R5C* treatment decreased the expression of B56γ, upregulated the levels of p‐AKT^Thr308^, p‐AKT^Ser473^, MMP2, and MMP9 in the si*2R5C* injection group (Figure 5H,I). In the liver tissues of nude mice harboured with HBx‐expressing MHCC97H xenograft tumours, considerably more migrated nodules were observed in the livers from the si*2R5C* group than those from the siNC group (Figures 5J and S4C). si*2R5C* treatment decreased the expression of B56γ, and increased the levels of p‐AKT^Thr308^, p‐AKT^Ser473^, MMP2, and MMP9 in the si*2R5C* injection group (Figure 5K,L). HE staining showed that the tumours migrated into the livers increased the prevalence of cancer nests associated with nodule formation and tumour angiogenesis (Figure 5L). These results demonstrated that B56γ silencing could aggravate the migratory and invasive hepatocarcinogenesis phenotype in HBV‐related HCC cells, both in vitro and in vivo, via targeting dephosphorylation regulation of the p‐AKT^Thr308/Ser473^‐MMP2/9 pathway.

### 3.6 Knockdown of B56γ promoted xenograft tumour growth and migration of HBx‐expressing HCC cells in vivo

**FIGURE 6:**
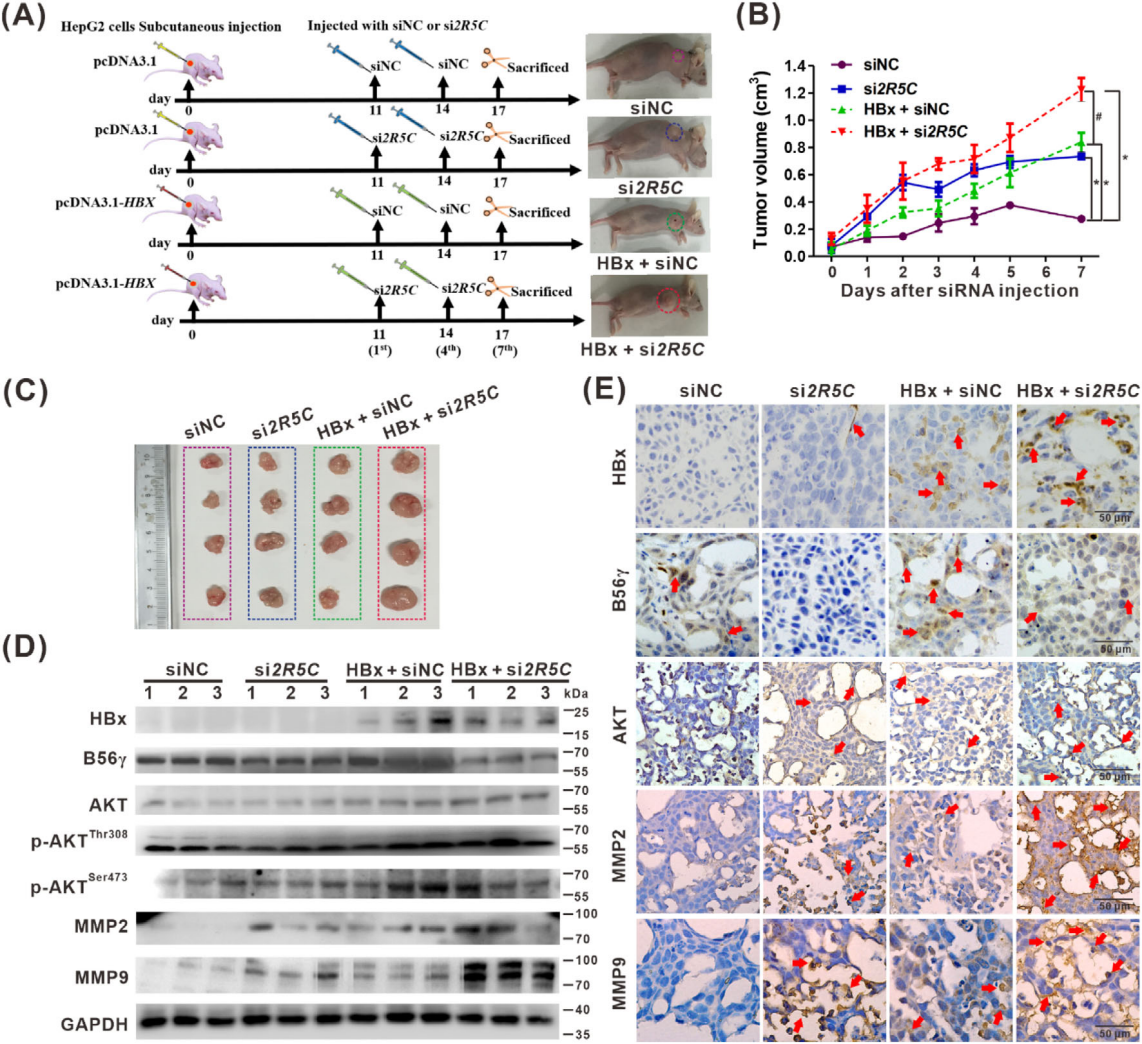
Knockdown of B56γ promoted xenograft tumour growth and migration of HBx‐expressing HCC cells in vivo. The xenograft tumours formed by HepG2‐pcDNA3.1‐*HBX* or HepG2‐pcDNA3.1 cells in BALB/c nude mice for 10 days. si*2R5C* or siNC was injected twice on the 11th and the 14th day (represented the 1st and the 4th day after the first injection, respectively). The tumours were excised on the 17th day (represented the 7th day after the first injection; *n* = 4). (A) Schematic diagram of experimental design for B56γ targeting intervention of xenograft tumours in BALB/c nude mice divided into four groups. (B) The growth curve of xenograft tumours was shown from day 0 to day 7 after the first injection. *P < 0.05, compared with the siNC control group. ^#^ *P* < 0.05, compared with the HBx‐expression group. (C) Xenograft tumour size. (D) The levels of HBx, B56γ, AKT, p‐AKT^Thr308^, p‐AKT^Ser473^, MMP2, and MMP9 proteins in the xenograft tumours were detected by Western blot. (E) Representative IHC images showed the expression level and distribution of HBx, B56γ, AKT, MMP2, and MMP9 in the xenograft tumour tissues. Arrows indicated the positive staining. Scale bars, 50 μm.

To further confirm the regulatory function of B56γ in hepatocarcinogenesis, we examined the role of B56γ in regulating dephosphorylation of AKT and inhibiting cell migration and invasion in nude mice model harboured HBx‐expressing HepG2 cell xenograft tumours (Figure 6A). Compared with the siNC control group, knockdown of B56γ with si*2R5C* injection promoted xenograft tumour growth with or without HBx‐expression (Figure 6B). The xenograft tumour volume (Figure 6B), size (Figure 6C), and weight (Figure S5) in the HBx‐expression group were much higher than those in the siNC control group, and the effect of promoting tumour growth was the most obvious in xenografts with HBx‐expression and knockdown of B56γ. In the xenograft tumours, compared with the siNC control group, HBx‐expression and knockdown of B56γ in the si*2R5C* group upregulated the expression of p‐AKT^Thr308/Ser473^ and tumour metastasis‐associated MMP2 and MMP9. These effects were enhanced in xenograft tumours with HBx‐expression and si*2R5C* injection, compared with the si*2R5C* injection group (Figure 6D). The IHC results confirmed these effects of B56γ knockdown on the increased levels of AKT, MMP2, and MMP9, and its synergistic upregulatory effect with HBx‐expression (Figure 6E). These results indicated that targeting knockdown of B56γ would promote HBx‐mediated HCC cell migration and invasion in vivo via decreasing the dephosphorylation of p‐AKT^Thr308/Ser473^. Therefore, we further explored the role of the inhibition or activation of B56γ on the regulation of p‐AKT^Thr308/Ser473^ dephosphorylation in HBx‐associated hepatocarcinogenesis.

### 3.7 B56γ targeted the dephosphorylation of p‐AKT to negatively regulate migration and invasion of HBx‐expressing HCC cells

**FIGURE 7:**
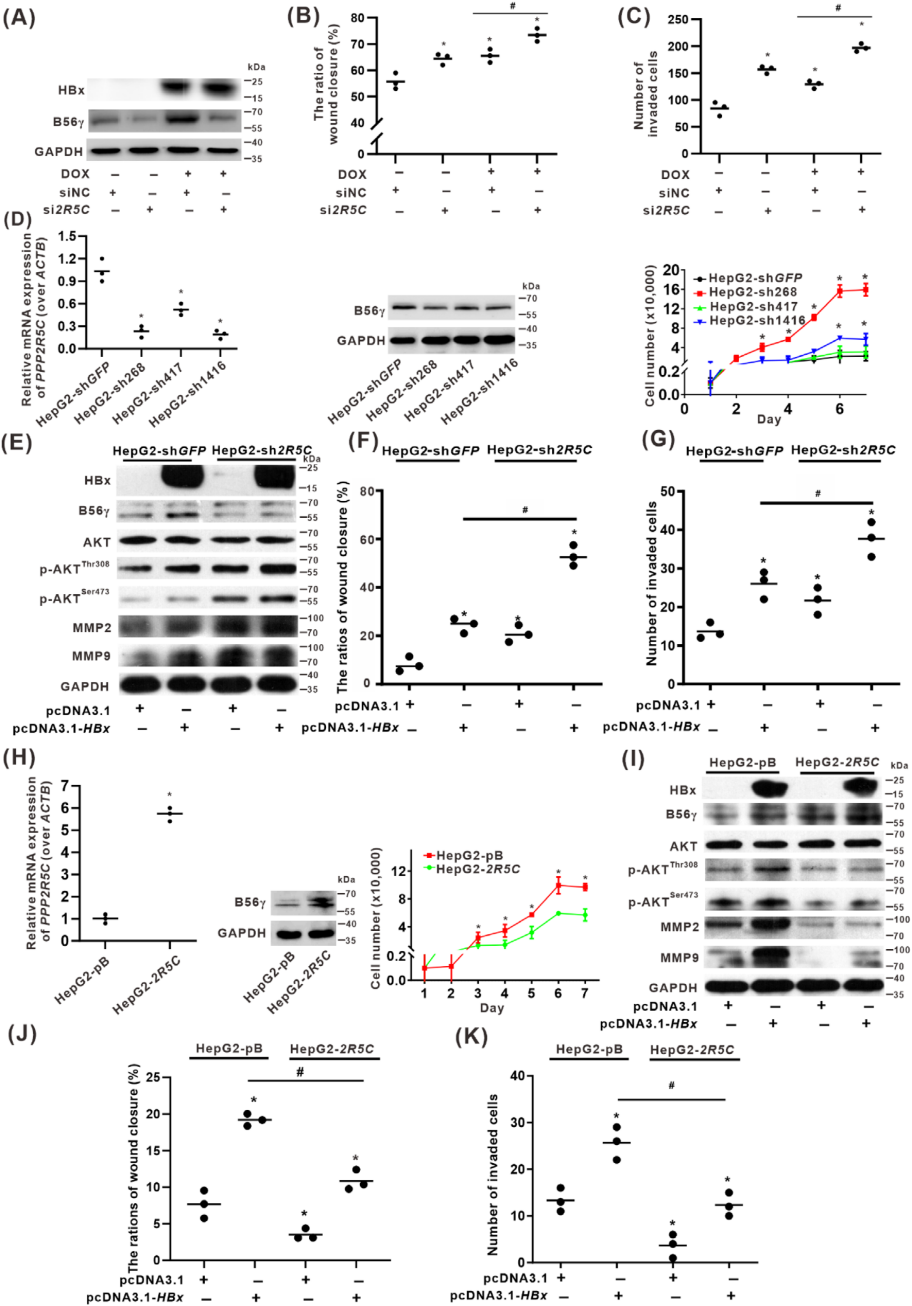
B56γ targeted the dephosphorylation of p‐AKT to negatively regulate migration and invasion of HBx‐expressing HCC cells. (A–C) HepG2‐Tet‐ON‐HBx cells were treated with or without DOX (1 μg/ml) and siNC or si*2R5C* for 24 h. (A) The levels of HBx, B56γ, AKT, p‐AKT^Thr308^, p‐AKT^Ser473^, MMP2, and MMP9 were detected by Western blot. (B,C) Wound healing and invasion assay were applied to evaluate migration and invasion ability. The relative quantitative ratio of wound closure (B) and the number of invaded cells (C) in each field of view are shown in the scatter plots. *P < 0.05, compared with the siNC group. ^#^ *P* < 0.05, compared with the HBx‐expressing group treated with DOX and transfected with siNC. (D–G) Stable B56γ‐knockdown HepG2‐sh*2R5C* cells were constructed with the pLKO.1 recombinant plasmid with shRNA targeting *PPP2R5C* (sh*2R5C*) at different coding sequence (CDS) sites. HepG2‐shGFP was served as a control cell. (D) Identification of B56γ knockdown in HepG2‐sh*2R5C* (sh268, sh417, and sh1416) cells (Left, Middle). The cell growth curves of HepG2‐sh*2R5C* and HepG2‐sh*GFP* cells (Right). *P < 0.05, compared with HepG2‐sh*GFP* cells on the same day. (E–G) HepG2‐sh268 cells were selected to represent the HepG2‐sh*2R5C* cells. (E) Western blots were conducted to evaluate the expression of proteins in cells with or without HBx‐expression transfected with pcDNA3.1‐*HBX*. (F,G) Wound healing assay (F) and invasion assay (G) were conducted. The relative quantitative ratio of wound closure (F) and the number of invaded cells (G) in each field of view are shown in the scatter plots. *P < 0.05, compared with the HepG2‐sh*GFP* cells. ^#^ *P* < 0.05, compared with the HepG2‐sh*GFP* cells transfected with pcDNA3.1‐*HBX* group. (H–K) Construction of HepG2‐*2R5C* cells for stable B56γ overexpression by transfection with the pBabe‐*2R5C* recombinant plasmid bearing *PPP2R5C* encoding sequence. HepG2‐pB was served as a control cell. (H) Identification of B56γ overexpression in HepG2‐*2R5C* cells (Left, Middle). The cell growth curves of HepG2‐*2R5C* and HepG2‐pB cells (Right). *P < 0.05, compared with HepG2‐pB cells on the same day. (I) Western blots were conducted to evaluate HBx, B56γ, AKT, p‐AKT^Thr308^, p‐AKT^Ser473^, MMP2, and MMP9 in HepG2‐*2R5C* and HepG2‐pB cells with or without HBx‐expression transfected with pcDNA3.1‐*HBX*. (J,K) Wound healing and invasion assay were applied to evaluate migration and invasion ability. The relative quantitative ratio of wound closure (J) and the number of invaded cells (K) in each field of view are shown in the scatter plots. *P < 0.05, compared with the HepG2‐pB cells. ^#^ *P* < 0.05, compared with the HepG2‐pB cells transfected with pcDNA3.1‐*HBX* group.

HBx‐associated cell migration and invasion depend on the phosphorylation of AKT at Thr308 and Ser473 in HBx‐expressing HCC cells. We constructed a B56γ‐knockdown expression model using si*2R5C* (Figure 7A). Interestingly, knockdown of B56γ promoted HBx‐induced cell migration and invasion (Figures 7B,C and S6A,B). To further demonstrate the impact of loss‐of‐function of B56γ, stable B56γ‐knockdown HepG2‐sh*2R5C* (sh268, sh417, and sh1416) cells were constructed and verified (Figure 7D). Compared with HepG2‐shGFP control cells, the growth rate of HepG2‐sh268 and ‐sh1416 cells were significantly increased, and the plate colony‐forming ability was increased (Figure S6C). HepG2‐sh268 cells with the fastest growth were used as the representative HepG2‐sh*2R5C* cells for follow‐up experiments (Figure 7D). The level of B56γ was decreased, and the expressions of p‐AKT^Thr308^, p‐AKT^Ser473^, MMP2, and MMP9 were increased in B56γ‐knockdown HepG2‐sh*2R5C* cells compared with the control group (Figure 7E). Moreover, inhibition of B56γ further enhanced p‐AKT^Thr308^ and p‐AKT^Ser473^, and MMP2 and MMP9 were induced by HBx‐expression (Figure 7E). In addition, knockdown of B56γ promoted HBx‐induced cell migration and invasion of HepG2‐sh*2R5C* cells compared with the control group (Figures 7F,G and S6D). It was suggested that genetic inhibition of the expression of B56γ was related to the HBx‐associated phenotype of hepatocarcinogenesis.

To explore the targeted effect of B56γ on the regulation of functional p‐AKT^Thr308/Ser473^ dephosphorylation in hepatocarcinogenesis, we further constructed stable B56γ‐overexpression HepG2‐*2R5C* cells for a targeted genetic intervention study (Figure 7H). Compared with the HepG2‐pBabe control cells, the growth rate of HepG2‐*2R5C* cells was significantly decreased (Figure 7H), while the plate colony‐forming ability was inhibited (Figure S6E). Moreover, B56γ was further increased in HBx‐expressing HepG2‐*2R5C* cells and significantly inhibited the increase in p‐AKT^Thr308/Ser473^, MMP2, and MMP9 induced by HBx‐expression (Figure 7I). In addition, consistent with the inhibition of cell growth and plate colony formation, the migration and invasion ability of HepG2‐*2R5C* cells were significantly reduced, while that of HBx‐expressing HepG2‐*2R5C* cells was significantly inhibited (Figures 7J,K and S6F). These results indicated that B56γ might especially target the dephosphorylation of p‐AKT^Thr308^ and p‐AKT^Ser473^ to negatively regulate the activation of p‐AKT signalling, while and gain‐of‐function of B56γ may represent a potential approach to inhibit the migratory and invasive phenotypes of HBx‐expressing HCC cells. Furthermore, the targeting blockade of HBx expression as the source intervention for HBV infection‐derived carcinogenesis should be further explored.

### 3.8 Intracellular expression of anti‐HBx mAb inhibited HBV‐related hepatocarcinogenesis phenotypes via blocking HBx expression in HCC cells

**FIGURE 8:**
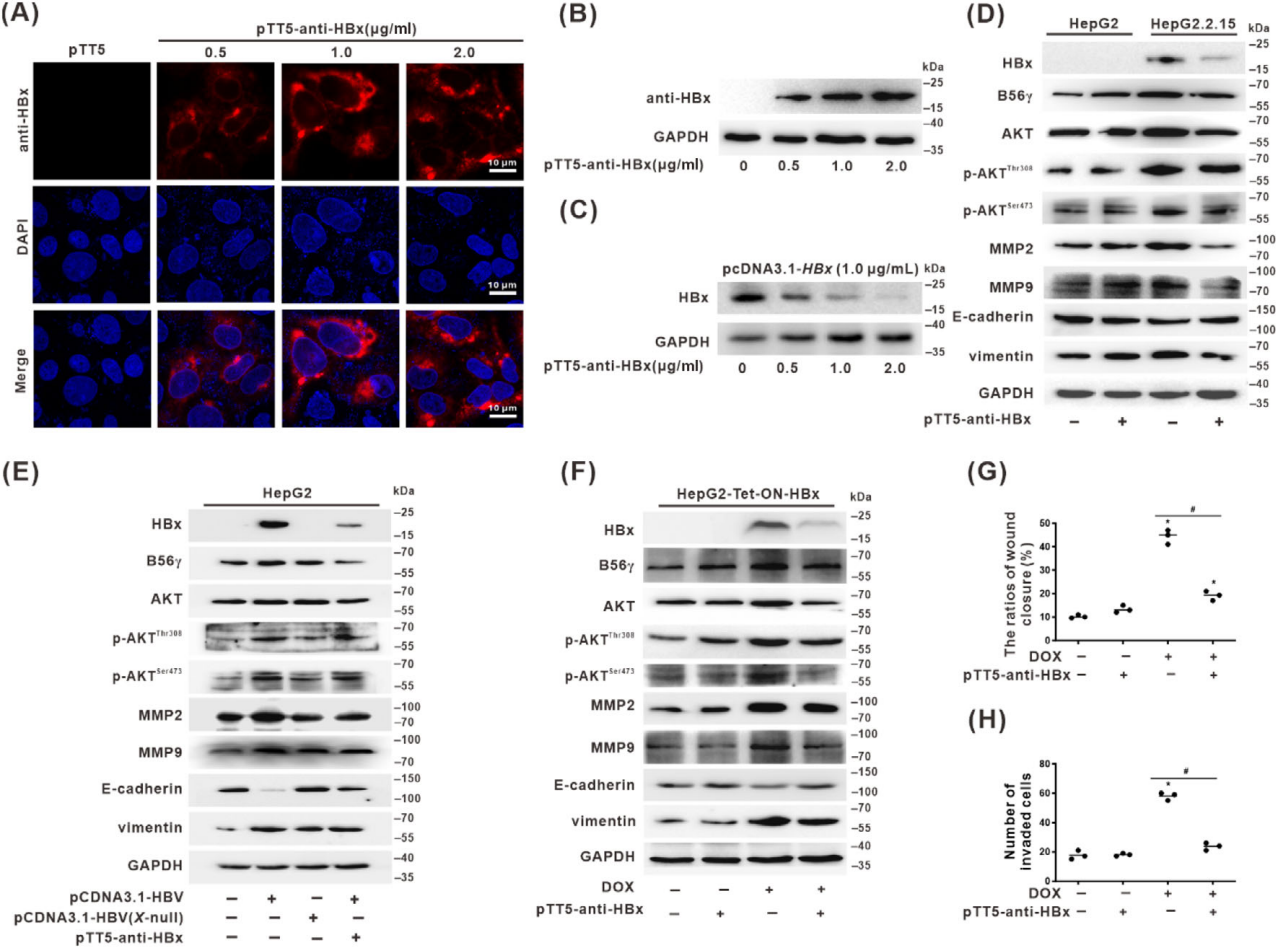
Intracellular expression of anti‐HBx targeting monoclonal antibody (mAb) inhibited HBV‐associated hepatocarcinogenesis phenotypes via blocking HBx expression in HCC cells. (A–C) The pTT5‐anti‐HBx(9D11) plasmid (0.5, 1, and 2 μg/ml) was transiently transfected into different HBx‐expressing cells for 24 h to establish the intracellular anti‐HBx mAb expression models. (A) Immunofluorescence representative images for evaluating the expression of anti‐HBx mAb (red) and DAPI (blue) staining in cells. Scale bars, 10 μm. (B) The expression level of anti‐HBx mAb in HepG2 cells was transiently transfected pTT5‐anti‐HBx plasmid in a dose‐dependent manner. (C) The expression level of HBx was detected by Western blot in HepG2 cells was transiently transfected pTT5‐anti‐HBx plasmid for 24 h after the pretreated with pcDNA3.1‐*HBX* (1 μg/ml). (D–H) The pTT5‐anti‐HBx(9D11) plasmid (1 μg/ml) was used for transient transfection for 24 h to establish the intracellular anti‐HBx mAb expression models in various HBx‐expressing HCC cells. The levels of HBx, B56γ, AKT, p‐AKT^Thr308^, p‐AKT^Ser473^, MMP2, MMP9, E‐cadherin, and vimentin proteins were detected by Western blot in HepG2 and HepG2.2.15 cells (D), and in pcDNA3.1‐HBV and pcDNA3.1‐HBV(*X*‐null) plasmids transfected HepG2 cells (E). (F‐H) HepG2‐Tet‐ON‐HBx cells for the HBx‐expressing model treated with DOX (1 μg/ml). (F) Western blot was conducted to evaluate the expression of HBx, B56γ, AKT, p‐AKT^Thr308^, p‐AKT^Ser473^, MMP2, MMP9, E‐cadherin, and vimentin. (G,H) Wound healing assay (G) and invasion assay (H) were applied to evaluate migration and HCC‐related invasion ability. The relative quantification ratio of wound closure (G) and the number of invaded cells (H) in each field of view are shown in the scatter plots. *P < 0.05, compared with the control group. ^#^ *P* < 0.05, compared with the HBx‐expressing group treated with DOX.

A previous study has reported that intracellularly expressed anti‐HBx antibody, a mAb generated by the fusion of a cell‐penetrating peptide (CPP) on the C‐terminus of the heavy chain of a potent mAb specific targeting to HBx, can promote the degradation of intracellular HBx.^20^ In the current study, this anti‐HBx mAb plasmid (pTT5‐anti‐HBx) was transiently transfected to produce the intracellular anti‐HBx mAb expression in HepG2 cells. It was shown that the plasmid could enter into and stably expressed in transfected HepG2 cells, where the intracellular anti‐HBx mAb was increased in dose‐dependent (Figures 8A,B and S7A). Our results showed that intracellular expression of anti‐HBx mAb exhibited significantly reduced the intracellular HBx level in HBx‐expressing HepG2 cells to explore the biological functions of anti‐HBx mAb specific targeting to block HBx (Figure 8C). The effects of anti‐HBx mAb on the inhibition of HBx‐associated hepatocarcinogenesis phenotypes were investigated in various HBV‐infected/HBx‐expressing HCC cells. In the HBV genome‐integrated HepG2.2.15 and pcDNA3.1‐HBV transfected HepG2 cells, HBx, B56γ, AKT, p‐AKT^Thr308^, p‐AKT^Ser473^, MMP2, MMP9, and vimentin proteins were increased, while the levels of E‐cadherin was decreased (Figure 8D). However, in pcDNA3.1‐HBV(*X*‐null) plasmid with HBV‐*X* gene deletion transfected HepG2 cells, the above results were suppressed, while the levels of B56γ, p‐AKT^Thr308^, p‐AKT^Ser473^ were decreased with the inhibition of intracellular HBx level (Figure 8E).

Moreover, in the HepG2.2.15 cells, HBV‐genome transfected HepG2 cells, and DOX‐treated HepG2‐Tet‐ON‐HBx cells treated with pTT5‐anti‐HBx plasmids, we found that intracellular product of anti‐HBx mAb significantly suppressed the HBx‐expression level in HBV‐related HCC cells, while followed by the inhibition of p‐AKT^Thr308/Ser473^, as well as the downregulation of MMP2, MMP9, and vimentin expression, and the increased levels of E‐cadherin (Figure 8D–F). This intracellular anti‐HBx mAb counteracted the effect of HBx‐expression in promoting of cell migration and invasion (Figures 8G,H and S7B,C). These results indicated that the anti‐HBx antibody may have chemopreventive applications and could control the migration and invasion of HBx‐expressing HCC cells through the activated B56γ‐mediated dephosphorylation of p‐AKT^Thr308/Ser473^. Moreover, targeting blockade of intracellular HBx was a functional approach to regulate the HBV/HBx‐associated hepatocarcinogenesis phenotypes via B56γ acting on the p‐AKT ^Thr308/Ser473^/MMPs/EMT pathway.

## 4 DISCUSSION

Hepatitis viruses are associated with diseases that cause liver injury, especially hepatocarcinogenesis.^28^, ^29^ HBV infection leads to chronic hepatitis and cirrhosis, contributing to hepatocarcinogenesis through direct and indirect mechanisms. The HBx protein encoded by the HBV *X* gene is important for HBV cccDNA transcription and viral replication, which plays a key role in HBV‐related HCC.^30^ Therefore, understanding the mechanism of HBV‐related HCC will provide strategies and opportunities for the multipattern prevention and control of HCC. In the current study, our results demonstrated that HBx‐expression induced the phosphorylation of specific AKT sites (p‐AKT^Thr308/Ser473^) and B56γ involved in mediating the migration and invasion phenotypes of HCC cells. However, genetic activation of B56γ mediated the dephosphorylation of p‐AKT^Thr308/Ser473^ to regulate HBV‐associated hepatocarcinogenesis's migration and invasion phenotypes in vitro and in vivo. A specific blockade of HBx‐expression via plasmid‐mediated targeting of intracellular anti‐HBx mAb production and genetic activation of B56γ would help target the p‐AKT^Thr308/Ser473^‐MMP2/9 signalling axis to mediate the multipattern chemoprevention and intervention in HBx‐associated hepatocarcinogenesis and HBV‐related HCC.

Many studies have shown that B56γ is an antitumour protein, whereas HBx is a pro‐tumour protein.^31^, ^32^, ^33^ This study focused on the role of B56γ and HBx expression in the migration and invasion phenotypes during hepatocarcinogenesis and screened for the regulation of AKT phosphorylation/dephosphorylation associated with B56γ and HBx expression. Therefore, we hypothesized that the dephosphorylation mechanism of B56γ on specific sites of p‐AKT^Thr308/Ser473^ is involved in the HBx‐related hepatocarcinogenesis signalling pathways in HCC cells. A study in HBV‐positive patient liver samples also demonstrated that AKT levels were elevated in HBV‐related HCC.^34^ It has been reported that regulating PTM, especially the balance of phosphorylation and dephosphorylation, is involved in cellular activities. Abnormal alterations in phosphorylation and the disruption of dephosphorylated homeostasis result in diseases such as cancer.^35^ In this study, we found that HBx expression promoted the migration and its related invasion in HCC cells via a mechanism related to AKT expression and its modification of functional p‐AKT^Thr308^ and p‐AKT^Ser473^ status. Similar to our results, Han et al. recently reported that AMPK fully activated AKT by enhancing its phosphorylation at Thr308 and Ser473 and, as a result, promoted cellular migration in breast cancer MDA‐MB‐231 cells.^36^ In addition, Liu H et al. showed that HBx promoted hepatoma cell invasion and metastasis by stabilizing Snail protein to enhance the expression of p‐AKT^Ser473^ by activating the PI3K/AKT/GSK‐3β signal pathway in Huh7 cells.^37^ In our study, to investigate the role of regulatory dephosphorylation of p‐AKT^Thr308/Ser473^ in hepatocarcinogenesis, the HBx‐Tg mice and a variety of HBV/HBx expression in vivo and in vitro experimental models have been established. Our results demonstrated that the levels of phosphorylated AKT (p‐AKT) at Thr308 (p‐AKT^Thr308^) and Ser473 (p‐AKT^Ser473^), which are essential for AKT pathway activation, were upregulated with the increased expression of HBx in vivo and in vitro. We further constructed the HCC cell models to mimic the dephosphorylation of AKT^T308A^ and AKT^S473A^. These results showed that dephosphorylation of p‐AKT^Thr308/Ser473^ inhibited the migration and invasion of HBx‐expressing HCC cells.

Our previous study found that B56γ, a protein phosphatase to target dephosphorylation, regulated cell cycle arrest and apoptosis in HBx‐expression hepatocytes.^12^ Functionally, PP2A has been studied for its role in tumour suppression via tumour metastasis signalling.^31^ Chen et al. reported that suppressing B56γ expression by Simian virus 40 small t antigen (SV40 ST) conferred HEK epithelial cell transformation and overexpressed B56γ attenuated ST‐induced tumorigenicity.^38^ Recently, Guo B et al. identified a micropeptide, CIP2A binding peptide (CIP2A‐BP), encoded by LINC00665, which can directly bind the tumour oncogene CIP2A to replace the B56γ subunit. The resulting PP2A activity, which inhibits the PI3K/AKT/NF‐κB pathway, led to the inhibition of tumour invasion and metastasis via decreasing the expression of MMP2, MMP9, and Snail in MDA‐MB‐231 cells.^39^ Moreover, He et al. reported that PP2A activation with SMAPs downregulated the levels of p‐AKT^Thr308^ and p‐AKT^Ser473^ and suppressed cell viability and cell cycle progression in Huh7 cells.^40^ In this study, we provided the first evidence that B56γ specifically targeted p‐AKT as a substrate to regulate the dephosphorylation of p‐AKT^Thr308^ and p‐AKT^Ser473^ and inhibit the MMP2/9‐associated metastasis phenotype of HBx‐expressing HCC cells in vitro and in vivo. So far, no specific agonist targeting the B56γ mediates PP2A‐B56γ/AC holoenzyme activation, although there is a specific activator for the PP2A‐B56α and PP2A‐B56ε holoenzyme. Therefore, selective activation of the PP2A holoenzyme, depending on the specific targeting activator for different PP2A‐B subunits, may be a potential strategy for the intervention and control of liver injuries and HBV/HBx‐related hepatocarcinogenesis.

Consistent with our results, Xi et al. suggested that PP2A regulatory subunit B56 could increase HBV core protein C‐terminal domain dephosphorylation and decrease nuclear HBV episomes, thereby inhibiting multiple stages of HBV replication.^11^ Xiao et al. demonstrated that PP2A protein phosphatase agonist d‐erythro‐Sphingosine could attenuate microcystins‐LR and C‐terminally truncated HBx‐induced the migration and invasion of HepG2 cells via regulating the dephosphorylation of PP2A/p‐p38 MAPK^Thr180/Tyr182^ axis.^41^ These studies found that although targeted activation of PP2A was found to rescue the malignant phenotype of HBx‐expressing cells, the expression level of PP2A protein itself was not detected, which needs further investigation. Surprisingly, we found that the HBx increased p‐AKT^Thr308/Ser473^ to drive HCC and then, at the same time, upregulated B56γ in the HBx‐expression cell model. We speculate that induction of the dephosphorylation function of B56γ might be a potentially novel pathway for feedback regulation of HBx‐related hepatocarcinogenesis. The upregulation of B56γ might be a dephosphorylation defence mechanism caused by HBX‐induced cellular stress, a new question that remains to be solved.

Moreover, in the current study, we used the HBx‐Tg mouse model to explore the pathogenesis of HBV infection‐related HCC. Different HBV mouse models (e.g., human‐mouse liver chimeric mice) developed to study infection and pathogenesis still have certain limitations.^42^, ^43^ Therefore, combining in vitro and in vivo models of HBx‐expressing cells and HBx‐Tg mice in this study, we further rigorously evaluated the applicability of these mouse models to the exploration of the mechanisms by which HBx‐associated hepatocarcinogenesis phenotypes, which will help provide important insights for the development of new therapeutic strategies for the treatment of HBV‐related HCC.

Identifying a specific function of HBx that might be targeted intervention is challenging. Slagle BL et al. highlighted that it is important to consider targeting to HBx for the prevention of HBV‐associated liver diseases due to the critical role of HBx in HBV replication and cell survival.^44^ Despite the successful development of preventive HBV vaccines that have effectively reduced new cases of HBV infection globally, hundreds of millions of people infected with HBV require more effective therapies.^45^ In recent years, mAbs have been shown to have a therapeutic role for infectious diseases and cancer.^46^ Zhang et al. reported that the intracellular expression of anti‐HBx mAb with a CPP could reduce HBx via Fc binding receptor tripartite motif containing‐21 (TRIM21)‐mediated protein degradation and could significantly suppress HBV transcription, replication, and viral antigen production both in vitro and in vivo.^20^ Furthermore, our previous results have suggested that the produced intracellular anti‐HBx mAb could reduce the cancer stemness characteristics of liver cancer associated with HBx‐induced B‐cell lymphoma 2/adenovirus E1B interacting 19 kDa‐interacting protein 3‐like (BNIP3L)‐dependent mitophagy and glycolysis metabolism reprogramming in HCC cells.^47^ In the current study, we further used this recombinant plasmid (pTT5‐anti‐HBx) to verify that the production of intracellular anti‐HBx mAb could block HBx expression and its induced B56γ‐mediated dephosphorylation activity, which, in turn, could inhibit the HBV‐associated migration and invasion of HBx‐expressing HCC cells. Our results showed a feasible strategy for inhibiting the bioeffects of HBx‐expression using a specific intracellular anti‐HBx antibody.

## 5 CONCLUSIONS

**FIGURE 9:**
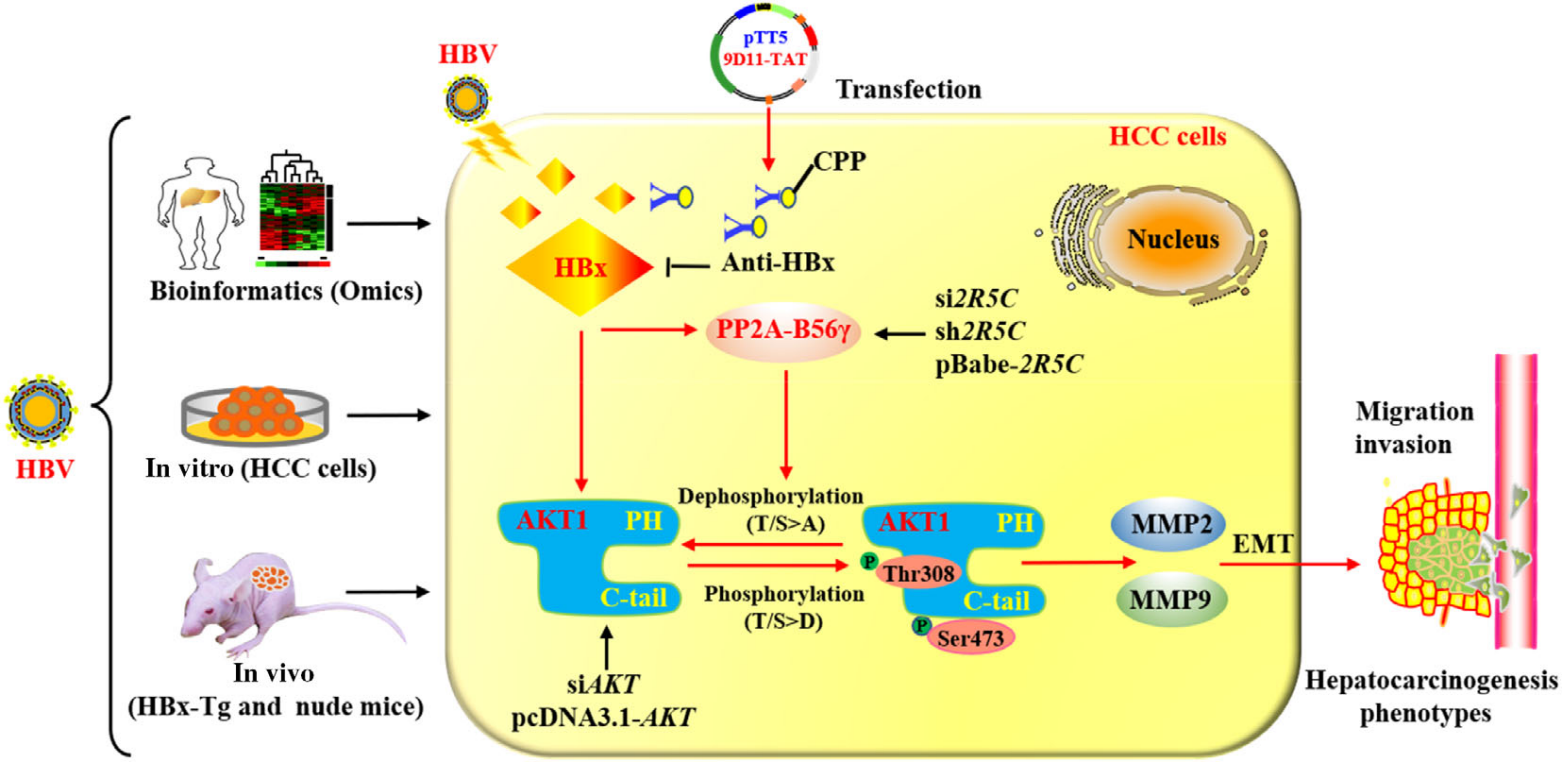
Schematic diagram of PP2A‐B56γ mediated the dephosphorylation of p‐AKT^Thr308/Ser473^ in HBx‐expressing HCC cells to regulate the migration and invasion phenotypes of HBV/HBx‐related hepatocarcinogenesis. In the current study, HBx‐expression induced the phosphorylation of specific AKT sites (p‐AKT^Thr308/Ser473^) involved in mediating the migration and invasion phenotypes of HCC cells. The inducible upregulation of B56γ mediated the dephosphorylation of p‐AKT^Thr308/Ser473^ in HBx‐expressing HCC cells. Specific blockade of HBx‐expression via pTT5‐anti‐HBx plasmid‐mediated targeting intracellular anti‐HBx mAb production and genetic activation of B56γ would help target the p‐AKT^Thr308/Ser473^‐MMP2/9 signalling axis to mediate the multipattern chemoprevention and intervention in HBV/HBx‐related hepatocarcinogenesis.

In conclusion, HBV/HBx induced the phosphorylation of specific AKT functional sites (Thr308 and Ser473) involved in cell migration and invasion in HBV‐related hepatocarcinogenesis. PP2A‐B56γ inhibited the p‐AKT^Thr308/Ser473^‐dependent hepatocarcinogenesis via a specific dephosphorylation regulatory mechanism, while the genetic intervention of *PPP2R5C* function mediated the targeted modulation of p‐AKT‐MMP2/9‐EMT‐mediated HCC cell phenotypes. Intracellular anti‐HBx mAb‐mediated specific blockade of HBx expression and its induced activation of B56γ would help target the B56γ‐p‐AKT^Thr308/Ser473^‐MMP2/9 signalling axis to mediate the intervention in HBx‐associated hepatocarcinogenesis (Figure 9). Altogether, our findings indicated that B56γ might be a potential therapeutic target and that anti‐HBx mAb may be the multipattern chemoprevention and therapeutical strategy for HBV‐related hepatocarcinogenesis.

## AUTHOR CONTRIBUTIONS

Zhong‐Ning Lin, Yu‐Chun Lin, and Xu Lin conceived and designed the study. Lin Che, Ze‐Bang Du, Wei‐Hua Wang, Jia‐Shen Wu, and Tun Han carried out experiments and analysed the data. Yuan‐Yuan Chen, Pei‐Yu Han, Zhao Lei, Xiao‐Xuan Chen, Yun He, and Ling Xu participated in statistical analyses and data interpretation. Lin Che, Ze‐Bang Du, Xu Lin, Zhong‐Ning Lin, and Yu‐Chun Lin drafted and revised the manuscript. All authors read and approved the final manuscript.

## CONFLICT OF INTEREST

The authors declare that they have no competing interests.

## ETHICS STATEMENT

All participants provided written informed consent, and the research was approved by the Medical Ethics Committee of Xiamen University. All animal experiments were approved by the Experimental Animal Ethics Committee of Xiamen University.

## Supporting information


**Appendix S1** Supporting Information.

## Data Availability

The raw data used to analyze the genes co‐expression and bioinformatics are available in the TCGA database (https://portal.gdc.cancer.gov/) and GEO database (https://www.ncbi.nlm.nih.gov/geo/) (GSE83148 and GSE96440). Other data supporting this study's findings are available within the article or from the authors upon request.

## References

[1] Bray F , Ferlay J , Soerjomataram I , Siegel RL , Torre LA , Jemal A . Global cancer statistics 2018: GLOBOCAN estimates of incidence and mortality worldwide for 36 cancers in 185 countries. CA Cancer J Clin. 2018;68(6):394‐424. 30207593 10.3322/caac.21492

[2] Cheng AL , Hsu C , Chan SL , Choo SP , Kudo M . Challenges of combination therapy with immune checkpoint inhibitors for hepatocellular carcinoma. J Hepatol. 2020;72(2):307‐319. 31954494 10.1016/j.jhep.2019.09.025

[3] Foerster F , Gairing SJ , Muller L , Galle PR . NAFLD‐driven HCC: safety and efficacy of current and emerging treatment options. J Hepatol. 2022;76(2):446‐457. 34555422 10.1016/j.jhep.2021.09.007

[4] Yang JD , Hainaut P , Gores GJ , Amadou A , Plymoth A , Roberts LR . A global view of hepatocellular carcinoma: trends, risk, prevention and management. Nat Rev Gastroenterol Hepatol. 2019;16(10):589‐604. 31439937 10.1038/s41575-019-0186-y PMC6813818

[5] Yang JD , Roberts LR . Epidemiology and management of hepatocellular carcinoma. Infect Dis Clin North Am. 2010;24(4):899‐919. 20937457 10.1016/j.idc.2010.07.004 PMC3949429

[6] Chen YY , Lin Y , Han PY , et al. HBx combined with AFB1 triggers hepatic steatosis via COX‐2‐mediated necrosome formation and mitochondrial dynamics disorder. J Cell Mol Med. 2019;23(9):5920‐5933. 31282064 10.1111/jcmm.14388 PMC6714226

[7] Levrero M , Zucman‐Rossi J . Mechanisms of HBV‐induced hepatocellular carcinoma. J Hepatol. 2016;64(1 Suppl):S84‐S101. 27084040 10.1016/j.jhep.2016.02.021

[8] Ryu SH , Jang MK , Kim WJ , Lee D , Chung YH . Metastatic tumor antigen in hepatocellular carcinoma: golden roads toward personalized medicine. Cancer Metastasis Rev. 2014;33(4):965‐980. 25325987 10.1007/s10555-014-9522-4

[9] Ventura JJ , Nebreda AR . Protein kinases and phosphatases as therapeutic targets in cancer. Clin Transl Oncol. 2006;8(3):153‐160. 16648114 10.1007/s12094-006-0005-0

[10] Shi Y . Serine/threonine phosphatases: mechanism through structure. Cell. 2009;139(3):468‐484. 19879837 10.1016/j.cell.2009.10.006

[11] Xi J , Luckenbaugh L , Hu J . Multiple roles of PP2A binding motif in hepatitis B virus core linker and PP2A in regulating core phosphorylation state and viral replication. PLoS Pathog. 2021;17(1):e1009230. 33493210 10.1371/journal.ppat.1009230 PMC7861550

[12] He C , Qiu Y , Han P , et al. ER stress regulating protein phosphatase 2A‐B56gamma, targeted by hepatitis B virus X protein, induces cell cycle arrest and apoptosis of hepatocytes. Cell Death Dis. 2018;9(7):762. 29988038 10.1038/s41419-018-0787-3 PMC6037732

[13] Mazhar S , Taylor SE , Sangodkar J , Narla G . Targeting PP2A in cancer: combination therapies. Biochim Biophys Acta Mol Cell Res. 2019;1866(1):51‐63. 30401535 10.1016/j.bbamcr.2018.08.020 PMC6436821

[14] Seshacharyulu P , Pandey P , Datta K , Batra SK . Phosphatase: PP2A structural importance, regulation and its aberrant expression in cancer. Cancer Lett. 2013;335(1):9‐18. 23454242 10.1016/j.canlet.2013.02.036 PMC3665613

[15] Revathidevi S , Munirajan AK . Akt in cancer: mediator and more. Semin Cancer Biol. 2019;59:80‐91. 31173856 10.1016/j.semcancer.2019.06.002

[16] Umesalma S , Kaemmer CA , Kohlmeyer JL , et al. RABL6A inhibits tumor‐suppressive PP2A/AKT signaling to drive pancreatic neuroendocrine tumor growth. J Clin Invest. 2019;129(4):1641‐1653. 30721156 10.1172/JCI123049 PMC6436899

[17] He W , Yan X , Pan S . Amphetamine neurotoxicity in PC12 cells through the PP2A/AKT/GSK3beta pathway. Neurotox Res. 2018;34(2):233‐240. 29511968 10.1007/s12640-018-9880-8

[18] Bhatt V , Shi K , Salamango DJ , et al. Structural basis of host protein hijacking in human T‐cell leukemia virus integration. Nat Commun. 2020;11(1):3121. 32561747 10.1038/s41467-020-16963-6 PMC7305164

[19] Che L , Wu JS , Du ZB , et al. Targeting mitochondrial COX‐2 enhances chemosensitivity via Drp1‐dependent remodeling of mitochondrial dynamics in hepatocellular carcinoma. Cancers (Basel). 2022;14(3):821. 35159089 10.3390/cancers14030821 PMC8834292

[20] Zhang JF , Xiong HL , Cao JL , et al. A cell‐penetrating whole molecule antibody targeting intracellular HBx suppresses hepatitis B virus via TRIM21‐dependent pathway. Theranostics. 2018;8(2):549‐562. 29290826 10.7150/thno.20047 PMC5743566

[21] Zhuang Q , Zhou T , He C , et al. Protein phosphatase 2A‐B55delta enhances chemotherapy sensitivity of human hepatocellular carcinoma under the regulation of microRNA‐133b. J Exp Clin Cancer Res. 2016;35:67. 27074866 10.1186/s13046-016-0341-z PMC4831140

[22] Chen X , Calvisi DF . Hydrodynamic transfection for generation of novel mouse models for liver cancer research. Am J Pathol. 2014;184(4):912‐923. 24480331 10.1016/j.ajpath.2013.12.002 PMC3969989

[23] Ong CP , Lee WL , Tang YQ , Yap WH . Honokiol: a review of its anticancer potential and mechanisms. Cancers (Basel). 2019;12(1):48. 10.3390/cancers12010048 PMC7016989 31877856

[24] Khemlina G , Ikeda S , Kurzrock R . The biology of hepatocellular carcinoma: implications for genomic and immune therapies. Mol Cancer. 2017;16(1):149. 28854942 10.1186/s12943-017-0712-x PMC5577674

[25] Akula SM , Abrams SL , Steelman LS , et al. RAS/RAF/MEK/ERK, PI3K/PTEN/AKT/mTORC1 and TP53 pathways and regulatory miRs as therapeutic targets in hepatocellular carcinoma. Expert Opin Ther Targets. 2019;23(11):915‐929. 31657972 10.1080/14728222.2019.1685501

[26] Liu Y , Tong Z , Li T , et al. Hepatitis B virus X protein stabilizes amplified in breast cancer 1 protein and cooperates with it to promote human hepatocellular carcinoma cell invasiveness. Hepatology. 2012;56(3):1015‐1024. 22473901 10.1002/hep.25751

[27] Zhang S , Li J , Liu P , et al. Pygopus‐2 promotes invasion and metastasis of hepatic carcinoma cell by decreasing E‐cadherin expression. Oncotarget. 2015;6(13):11074‐11086. 25871475 10.18632/oncotarget.3570 PMC4484440

[28] El‐Serag HB . Epidemiology of viral hepatitis and hepatocellular carcinoma. Gastroenterology. 2012;142(6):1264‐1273. 22537432 10.1053/j.gastro.2011.12.061 PMC3338949

[29] Hou C , Hua Z , Xu P , et al. Estimating the prevalence of hepatitis B by wastewater‐based epidemiology in 19 cities in China. Sci Total Environ. 2020;740:139696. 32927529 10.1016/j.scitotenv.2020.139696

[30] Neuveut C , Wei Y , Buendia MA . Mechanisms of HBV‐related hepatocarcinogenesis. J Hepatol. 2010;52(4):594‐604. 20185200 10.1016/j.jhep.2009.10.033

[31] Nobumori Y , Shouse GP , Fan L , Liu X . HEAT repeat 1 motif is required for B56gamma‐containing protein phosphatase 2A (B56gamma‐PP2A) holoenzyme assembly and tumor‐suppressive function. J Biol Chem. 2012;287(14):11030‐11036. 22315229 10.1074/jbc.M111.334094 PMC3322884

[32] Moreno C , Ramachandran S , Ashby D , et al. Signaling and transcriptional changes critical for transformation of human cells by simian virus 40 small tumor antigen or protein phosphatase 2A B56γ knockdown. Cancer Res. 2004;64(19):6978‐6988. 15466190 10.1158/0008-5472.CAN-04-1150

[33] Lara‐Pezzi E , Gomez‐Gaviro M , Galvez B , et al. The hepatitis B virus X protein promotes tumor cell invasion by inducing membrane‐type matrix metalloproteinase‐1 and cyclooxygenase‐2 expression. J Clin Invest. 2002;110(12):1831‐1838. 12488433 10.1172/JCI200215887 PMC151651

[34] Golob‐Schwarzl N , Krassnig S , Toeglhofer AM , et al. New liver cancer biomarkers: PI3K/AKT/mTOR pathway members and eukaryotic translation initiation factors. Eur J Cancer. 2017;83:56‐70. 28715695 10.1016/j.ejca.2017.06.003

[35] Singh V , Ram M , Kumar R , Prasad R , Roy BK , Singh KK . Phosphorylation: implications in cancer. Protein J. 2017;36(1):1‐6. 28108801 10.1007/s10930-017-9696-z

[36] Han F , Li CF , Cai Z , et al. The critical role of AMPK in driving Akt activation under stress, tumorigenesis and drug resistance. Nat Commun. 2018;9(1):4728. 30413706 10.1038/s41467-018-07188-9 PMC6226490

[37] Liu H , Xu L , He H , et al. Hepatitis B virus X protein promotes hepatoma cell invasion and metastasis by stabilizing snail protein. Cancer Sci. 2012;103(12):2072‐2081. 22957763 10.1111/cas.12017 PMC7659259

[38] Chen W , Possemato R , Campbell KT , Plattner CA , Pallas DC , Hahn WC . Identification of specific PP2A complexes involved in human cell transformation. Cancer Cell. 2004;5(2):127‐136. 14998489 10.1016/s1535-6108(04)00026-1

[39] Guo B , Wu S , Zhu X , et al. Micropeptide CIP2A‐BP encoded by LINC00665 inhibits triple‐negative breast cancer progression. EMBO J. 2020;39(1):e102190. 31755573 10.15252/embj.2019102190 PMC6939193

[40] He X , Li M , Yu H , et al. Loss of hepatic aldolase B activates Akt and promotes hepatocellular carcinogenesis by destabilizing the Aldob/Akt/PP2A protein complex. PLoS Biol. 2020;18(12):e3000803. 33275593 10.1371/journal.pbio.3000803 PMC7744066

[41] Xiao C , Mei F , Ren G , et al. Synergistic effect of MC‐LR and C‐terminal truncated HBx on HepG2 cells and their effects on PP2A mediated downstream target of MAPK signaling pathway. Front Genet. 2020;15(11):537785. 10.3389/fgene.2020.537785 PMC7593820 33193609

[42] Yuan L , Jiang J , Liu X , et al. HBV infection‐induced liver cirrhosis development in dual‐humanised mice with human bone mesenchymal stem cell transplantation. Gut. 2019;68(11):2044‐2056. 30700543 10.1136/gutjnl-2018-316091 PMC6839735

[43] Du Y , Broering R , Li X , et al. In vivo mouse models for hepatitis B virus infection and their application. Front Immunol. 2021;12:766534. 34777385 10.3389/fimmu.2021.766534 PMC8586444

[44] Slagle BL , Bouchard MJ . Role of HBx in hepatitis B virus persistence and its therapeutic implications. Curr Opin Virol. 2018;30:32‐38. 29454995 10.1016/j.coviro.2018.01.007 PMC5988931

[45] Schweitzer A , Horn J , Mikolajczyk RT , Krause G , Ott JJ . Estimations of worldwide prevalence of chronic hepatitis B virus infection: a systematic review of data published between 1965 and 2013. Lancet. 2015;386(10003):1546‐1555. 26231459 10.1016/S0140-6736(15)61412-X

[46] Ebbers M , Hemmer CJ , Muller‐Hilke B , Reisinger EC . Immunotherapy and vaccination against infectious diseases. Wien Klin Wochenschr. 2020;2020:714‐720. 10.1007/s00508-020-01746-2 PMC7738774 33326055

[47] Chen YY , Wang WH , Che L , et al. BNIP3L‐dependent mitophagy promotes HBx‐induced cancer stemness of hepatocellular carcinoma cells via glycolysis metabolism reprogramming. Cancers (Basel). 2020;12(3):655. 10.3390/cancers12030655 PMC7139741 32168902

