# Supplementary material for: Intracellular antibody targeting HBx suppresses invasion and metastasis in hepatitis B virus‐related hepatocarcinogenesis via protein phosphatase 2A‐B56γ‐mediated dephosphorylation of protein kinase B
Source: Cell Prolif. 2022 Jul 10;55(11):e13304. doi: 10.1111/cpr.13304 (PMC9628248; doi:10.1111/cpr.13304)
Supplement: Supplementary file 1 — Appendix S1 Supporting Information. [file CPR-55-e13304-s001.docx]

**Supplementary material**

**Intracellular antibody targeting HBx suppresses invasion and metastasis in hepatitis B virus-related hepatocarcinogenesis via protein phosphatase 2A-B56γ-mediated dephosphorylation of protein kinase B**

Lin Che^1,^ *, Ze-Bang Du^1,^ *, Wei-Hua Wang^1,^ *, Jia-Shen Wu^1^, Tun Han^1^, Yuan-Yuan Chen^1,3^, Pei-Yu Han^1,4^, Zhao Lei^1^, Xiao-Xuan Chen^1^, Yun He^2^, Ling Xu^1^, Xu Lin^2, ✉^, Zhong-Ning Lin^1, ✉^, and Yu-Chun Lin^1, ✉^

^1^ State Key Laboratory of Molecular Vaccinology and Molecular Diagnostics, School of Public Health, Xiamen University, Xiamen, 361102, China.

^2^ Key Laboratory of Ministry of Education for Gastrointestinal Cancer, School of Basic Medical Sciences, Fujian Medical University, Fuzhou, 350004, China.

^3^ China CDC Key Laboratory of Environment and Population Health, National Institute of Environmental Health, Chinese Center for Disease Control and Prevention, Beijing, 100021, China.

^4^ Wuxi School of Medicine, Jiangnan University, Wuxi, 214122, China.

E-mail: CL: [1073773221@qq.com](mailto:1073773221@qq.com), DZB: [15155178530@163.com](mailto:15155178530@163.com), WWH: [1757331175@qq.com](mailto:1757331175@qq.com), WJS: [303642898@qq.com](mailto:303642898@qq.com), HT: [1620343536@qq.com](mailto:1620343536@qq.com), CYY: [chenyuanyuan@nieh.chinacdc.cn](mailto:chenyuanyuan@nieh.chinacdc.cn), HPY: [hpy106@126.com](mailto:hpy106@126.com), LZ: [leizhao@xmu.edu.cn](mailto:leizhao@xmu.edu.cn), CXX: [chen_xuan17@xmu.edu.cn](mailto:xuanchen_xuan17@xmu.edu.cn), HY: [hey607@126.com](mailto:hey607@126.com), XL: lingxu@xmu.edu.cn

* These authors contributed equally to this work

^✉^Corresponding author: Xu Lin, Key Laboratory of Ministry of Education for Gastrointestinal Cancer, School of Basic Medical Sciences, Fujian Medical University, Fuzhou, 350004, China. Zhong-Ning Lin, Yu-Chun Lin, State Key Laboratory of Molecular Vaccinology and Molecular Diagnostics, School of Public Health, Xiamen University, Xiang'an South Road, Xiamen, 361102, China.

Tel: +86 592 2880615;

Fax: +86 592 2881578.

E-mail: [linxu@mail.fjmu.edu.cn](mailto:linxu@mail.fjmu.edu.cn), [linzhn@xmu.edu.cn](mailto:linzhn@xmu.edu.cn), and [linych@xmu.edu.cn](mailto:linych@xmu.edu.cn).

**Supplementary figures**

**
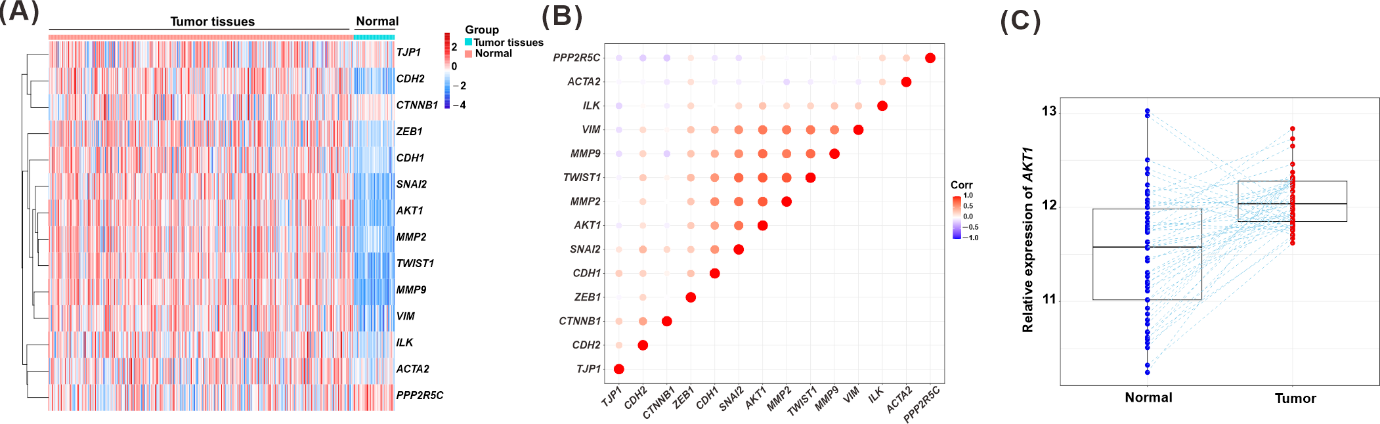
**

**Fig. S1. Cell migration and the AKT pathway were associated with hepatocarcinogenesis in bioinformatics analysis of HCC tissue.** (A, B) The Cancer Genome Atlas (TCGA) dataset in tumor and normal tissues samples from HCC patients. (A) The heatmap of DEGs in the HCC tumor tissues and the normal samples. The color bars on the top represented the experimental groups. The red bar represents HCC tumor tissues (n = 373), and the green bar represents non-neoplastic liver tissues (n = 50). The longitudinal clusters represented the related DEGs, which red indicating up-regulation and blue indicating down-regulation. (B) Pearson's correlation analysis was used for the correlationship between the relative expression levels of the indicated genes. (C) The trend relationship analysis for *AKT1* relative expression in 50 paired of normal and tumor tissues was shown.


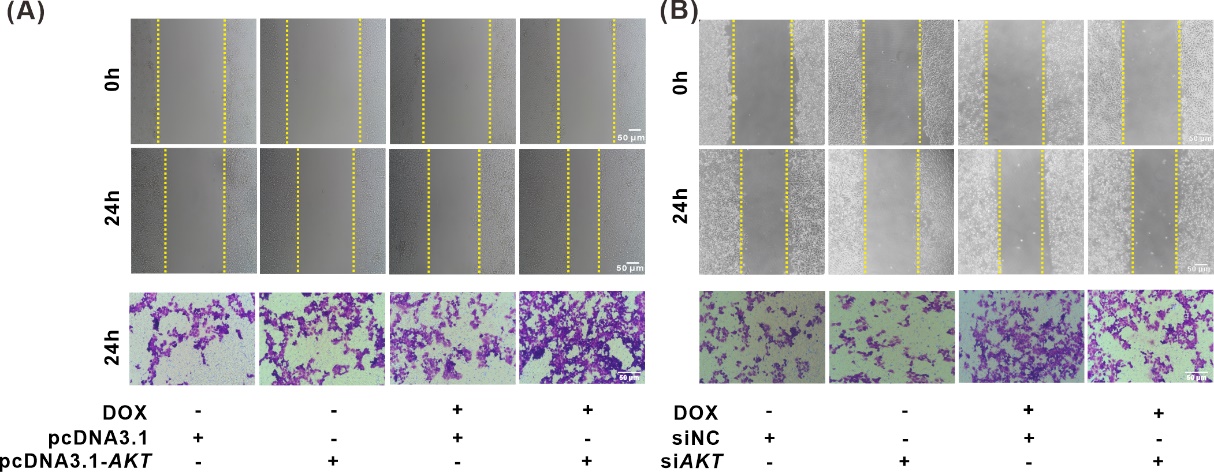


**Fig. S2. AKT** **was involved in regulating the migration and HCC-related invasion of HBx-expressing cells.** (A) AKT overexpression model was established in HBx-expressing HCC cells, in which HepG2-Tet-ON-HBx cells were treated with DOX (1 μg/ml) and transfected with pcDNA3.1 or pcDNA3.1-*AKT* for 24 h. Representative images of wound healing assay (Upper) and transwell invasion assay (Lower) were shown. Scale bar, 50 μm. (B) si*AKT* was used to knock down AKT expression in HBx-expressing cells, in which HepG2-Tet-ON-HBx cells were treated with DOX (1 μg/ml) and transfected with siNC or si*AKT* for 24 h. siNC was as the negative control (NC). Representative images of wound healing assay (Upper) and transwell invasion assay (Lower) were shown. Scale bar, 50 μm.


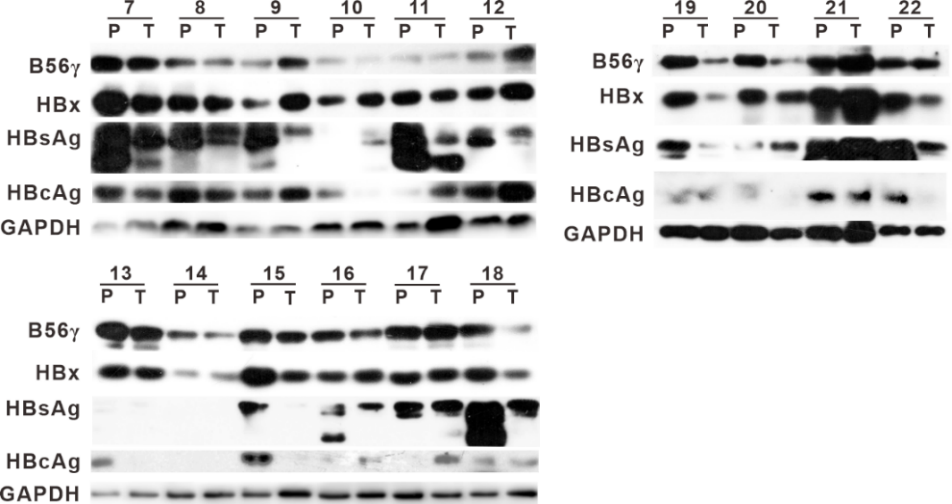


**Fig. S3.** **The levels of HBV-related proteins and B56γ in HBV-infected human HCC tumor tissues and adjacent peritumor tissues.** Levels of B56γ, HBx, HBsAg, and HBcAg proteins expression in 16 of 22 paired adjacent peritumor (P) and tumor (T) specimens from HCC patients were detected by Western blot.


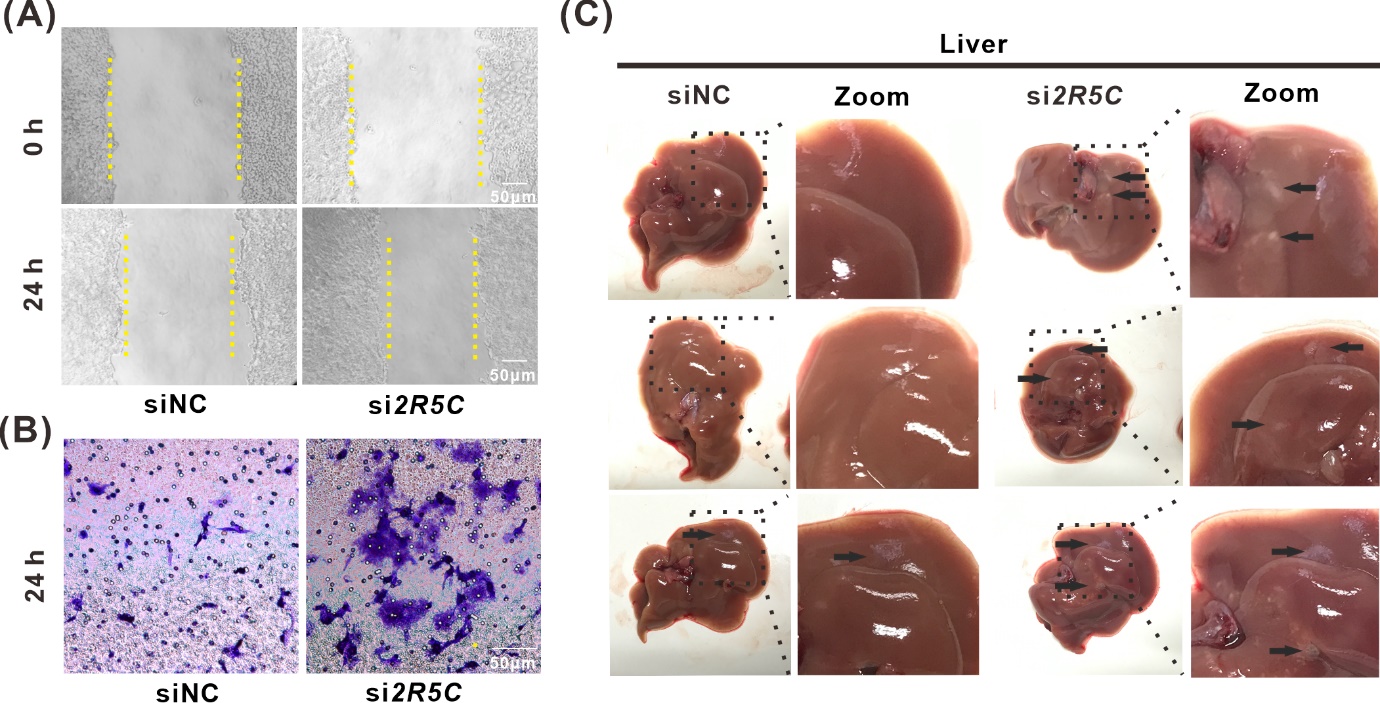


**Fig. S4. B56γ** **knockdown aggravated the HCC phenotypes of migration and invasion in HBV-associated MHCC97H cells.** MHCC97H cells, a HBV genome-integrated and highly metastatic HCC cell line, were used. MHCC97H cells were transfected with small interfering RNA targeting *PPP2R5C* gene (si*2R5C*) for 24 h to knock down the expression of PP2A-B56γ, while siNC was served as negative control (NC). Representative wound healing images (A) and transwell invasion cells images (B) were shown. Scale bar, 50 μm. (C) Excised livers from xenograft tumor-bearing mice were photographed to show big and detailed pictures (Zoom), while arrows indicated the HCC migrated nodules in the liver tissues.


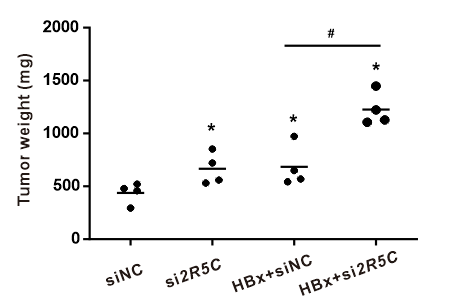


**Fig. S5. Knockdown of B56γ promoted xenograft tumors growth** **and migration of HBx-expressing HCC cells *in vivo*.** The xenograft tumors formed by HepG2-pcDNA3.1-*HBX* or HepG2-pcDNA3.1 cells in BALB/c nude mice for 10 days. si*2R5C* or siNC was injected twice on the 11^th^ and the 14^th^ day and the tumors were excised on the 17^th^ day. Xenograft tumor weight is shown in the scatter plot. n = 4. * *P* < 0.05, compared with the siNC control group. ^#^ *P* < 0.05, compared with the HBx-expression group.


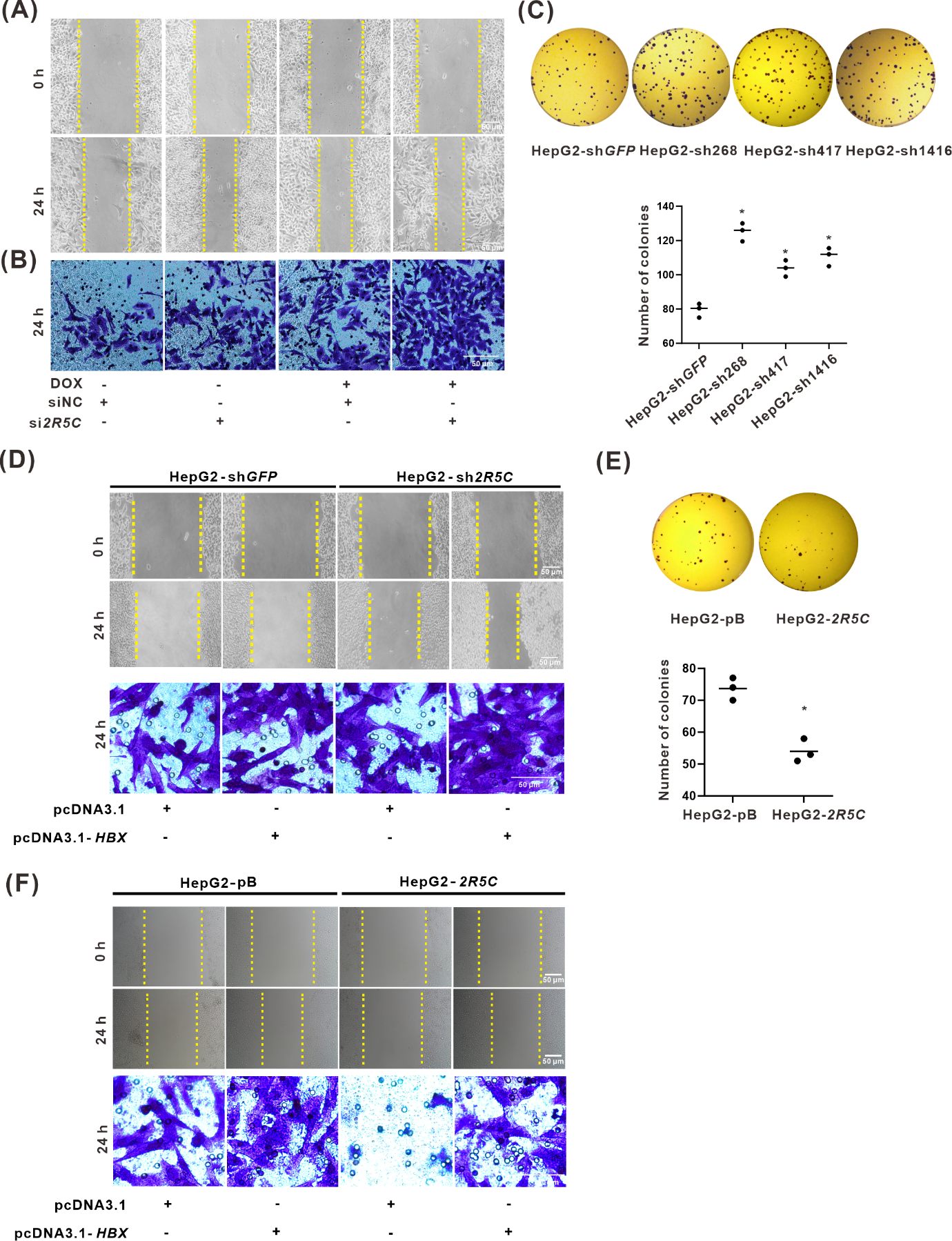


**Fig. S6. B56γ inhibited the migration and invasion phenotypes of HBx-expressing HCC cells.** (A, B) HepG2-Tet-ON-HBx cells were treated with or without DOX (1 μg/ml) and siNC or si*2R5C* for 24 h. (A) Representative wound healing images and (B) transwell invasion cells images were shown. Scale bar, 50 μm. (C) The self-renewal capacity was shown in photographs (Upper) and the scatter plot (Lower) for the three HepG2-sh*2R5C* cell lines (sh268, sh417, and sh1416) and the HepG2-sh*GFP* cells as evaluated using the cellular colony formation assay. * *P* < 0.05, compared with HepG2-sh*GFP* cells. (D) HepG2-sh*2R5C* and HepG2-sh*GFP* cells with or without HBx-expression transfected with pcDNA3.1-*HBX*. Representative wound healing images (Upper) and transwell invasion cells images (Lower) were shown. Scale bar, 50 μm. (E) The self-renewal capacities of HepG2-*2R5C* cells and HepG2-pB cells were evaluated using the cellular colony formation assay, as shown in photographs (Upper) and the scatter plot (Lower). * *P* < 0.05, compared with HepG2-pB cells. (F) HepG2-*2R5C* and HepG2-pB cells with or without HBx-expression transfected with pcDNA3.1-*HBX*. Representative wound healing images (Upper) and transwell invasion cells images (Lower) were shown. Scale bar, 50 μm.


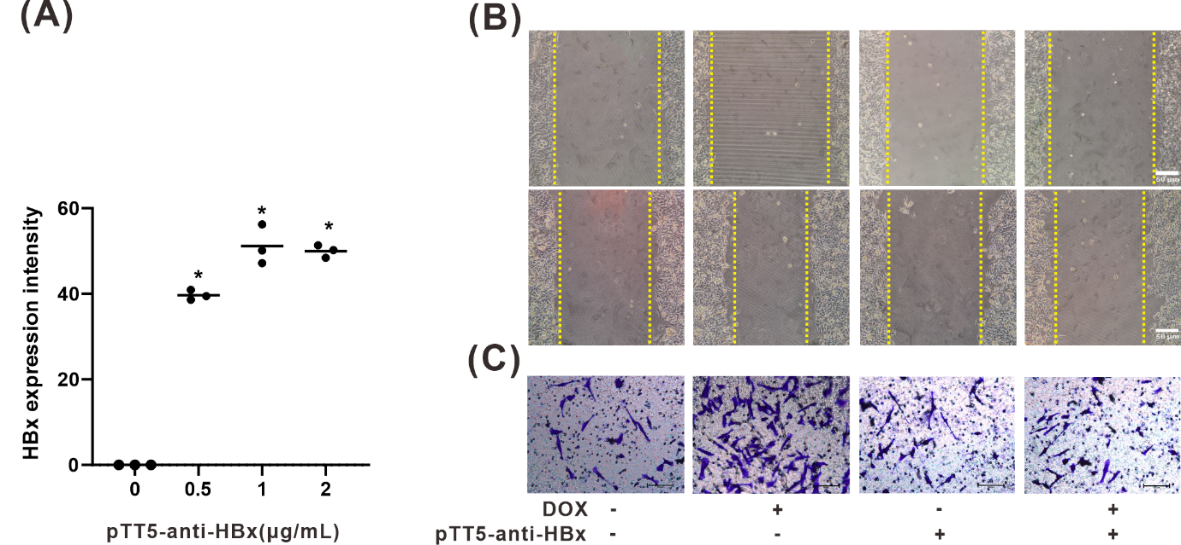


**Fig. S7. Anti-HBx inhibited the migration and invasion phenotypes of HBx-expressing HCC cells.** (A) The pTT5-anti-HBx(9D11) plasmid (0.5, 1, and 2 μg/ml) was transiently transfected into different HBx-expressing cells for 24 h to establish the anti-HBx mAb intracellular expression models. Quantification analysis of the intracellular HBx-expression is shown in the scatter plot. n = 3 in each group. * *P* < 0.05, compared with the control group. (B, C) HBx-expressing cells were transfected by pTT5-anti-HBx plasmid (1 μg/ml) for expressing intracellular anti-HBx for 24 h. (B) Representative wound healing images and (C) transwell invasion cells images were shown. Scale bar, 50 μm.

**Table S1 Primers for site-directed mutagenesis of *AKT***

| Mutant plasmids | Primers* |
| --- | --- |
| T308D | FP: 5’- **GA**CTTTTGCGGCACACCTGAGTACCTGG-3’  RP: 5’- CTTCATGGTGGCACCGTCCTTGATCCCCT-3’ |
| T308A | FP: 5’- **G**CCTTTTGCGGCACACCTGAGTACCTGG-3’  RP: 5’- CTTCATGGTGGCACCGTCCTTGATCCCCT-3’ |
| S473D | FP: 5’-**GA**CTACTCGGCCAGCGGCACGGCCTGA-3’  RP: 5’-GAACTGGGGGAAGTGGGGCCTGCGCTC-3’ |
| S473A | FP: 5’-**G**CCTACTCGGCCAGCGGCACGGCCTGA-3’  RP: 5’-GAACTGGGGGAAGTGGGGCCTGCGCTC-3’ |

* Indicated bases of mutant sites were underlined and bolded.

Abbreviations: A: Alanine; D: Aspartate; FP: Forward primer; RP: Reverse primer; S: Serine; T: Threonine.

**Table S2. Primers for establishment of stable B56γ-knockdown and -overexpression cell lines**

| Primer name | Sequence (5' - 3') |
| --- | --- |
| *2R5C*-sh268 FP | CCGGTCCAGAAGTTACGTCAGTGTTCTCGAGAACACTGACGTAACTTCTGGATTTTTG |
| *2R5C*-sh268 RP | AATTCAAAAATCCAGAAGTTACGTCAGTGTTCTCGAGAACACTGACGTAACTTCTGGA |
| *2R5C*-sh417 FP | CCGGCCAGAAGTAGTCCATATGTTTCTCGAGAAACATATGGACTACTTCTGGTTTTTG |
| *2R5C*-sh417 RP | AATTCAAAAACCAGAAGTAGTCCATATGTTTCTCGAGAAACATATGGACTACTTCTGG |
| 2R5C-sh1416 FP | CCGGCGGGAAGAAGCATGGGTTAAACTCGAGTTTAACCCATGCTTCTTCCCGTTTTTG' |
| *2R5C*-sh1416 RP | AATTCAAAAACGGGAAGAAGCATGGGTTAAACTCGAGTTTAACCCATGCTTCTTCCCG |
| *2R5C*+109*Bam*PF | GCCGGATCCATGTTGACATGTAATAAAGCGGGC |
| *2R5C*+1683*FlagSal*RP | GAGTCGACTTACTTATCGTCGTCATCCTTGTAATCGCGGCCGTCCTGGGAGG |

Abbreviations: *2R5C*: *PPP2R5C*; *Bam*: *BamHI*; FP: Forward primer; RP: Reverse primer; *Sal*: *SalI*; sh: short hairpin.
